# Large-scale genomic analysis reveals the distribution and diversity of type VI secretion systems in *Escherichia coli*

**DOI:** 10.1128/msystems.00105-25

**Published:** 2025-06-18

**Authors:** Kristina Nesporova, Bart Steemans, Sander K. Govers

**Affiliations:** 1 Department of Biology, KU Leuven 26657, Leuven, Flanders, Belgium; Northwestern University Feinberg School of Medicine, Chicago, Illinois, USA

**Keywords:** type VI secretion system, large-scale bioinformatics, *Escherichia coli*, ExPEC, IPEC

## Abstract

The type VI secretion system (T6SS) is a versatile nanomachine that injects effectors into target cells, playing a role in bacterial competition and virulence. While widespread in Gram-negative bacteria, T6SS prevalence varies across species and strains, and its distribution in *Escherichia coli* remains underexplored despite it being an important intestinal (IPEC) and extraintestinal (ExPEC) pathogen. Here, we examined the prevalence of T6SS subclasses (T6SS^i^) across 131,610 *E. coli* genomes, which we annotated for clinical relevance. T6SS genes were identified while focusing on the three subclasses present in *E. coli:* T6SS^i1^, T6SS^i2^, and T6SS^i4b^. Across phylogenetic groups, T6SS^i1^ was broadly present, while T6SS^i2^ showed associations with B1, B2, and G, and T6SS^i4b^ was rare. T6SS^i1^ was primarily associated with IPEC, and T6SS^i2^ with ExPEC. Even clearer patterns emerged at the sequence type (ST) level. For example, both the most dominant ExPEC and IPEC ST (ST131 and ST11, respectively) displayed niche-specific trends, with non-complete T6SSs being more associated with humans. We also evaluated the co-occurrence of T6SSs with other virulence-associated genes (VAGs) and multidrug resistance (MDR). This analysis confirmed the association of T6SS^i1^ and T6SS^i2^ with IPEC- and ExPEC-associated VAGs, respectively, and revealed a negative correlation between complete T6SS^i^ subclasses and MDR. Finally, we demonstrated how the presence of different T6SSs and VAGs can be used to examine and distinguish IPEC- and ExPEC-associated genomes. Together, our work provides a comprehensive overview of the diversity of T6SSs across *E. coli*, shedding more light on their potential contribution to pathogenicity in this species.

**IMPORTANCE:**

Our study represents a large-scale analysis of T6SSs across one of the most comprehensive collections of *E. coli* genomes to date. In doing so, we updated several misconceptions on T6SSs distribution and other genomic properties of *E. coli* strains, which originated from smaller-scale studies and were subsequently extrapolated in the literature. This includes the prevalence and distribution of T6SS^i^ subclasses across phylogenetic groups (e.g., T6SS^i2^ is not prevalent in phylogroup D), the association of specific virulence factors with IPEC and/or ExPEC (e.g., hemolysin A is more often associated with IPEC and not a typical ExPEC characteristic), and characteristics of pathogenic STs (e.g., ST131 displays distinct genomic properties based on its environmental niche). As such, this study not only advances our understanding of T6SS in *E. coli* but also serves as a valuable resource for future studies on the clinical relevance and distribution of other genetic elements.

## INTRODUCTION

*Escherichia coli* is one of the most well-known and studied organisms, serving as a valuable bacterial model species and laboratory workhorse for molecular biology. While most *E. coli* laboratory strains do not possess pathogenic properties, *E. coli* also exhibits extreme intra-species diversity due to its rich and variable accessory genome (1, 2). This leads to some *E. coli* strains occurring as gut commensals (3, 4), while others act as (opportunistic) pathogens that can cause either intestinal infections or extra-intestinal infections (in both humans and animals). Intestinal pathogenic *E. coli* (IPEC) infections display a diverse range of clinical manifestations, from mild diarrhea to potentially deadly hemorrhagic uremic syndrome (5, 6). Extraintestinal pathogenic *E. coli* (ExPEC) are responsible for the majority of urinary tract infections (UTIs) in humans and a large part of bloodstream infections (BSIs), which can result in sepsis (7, 8). Within these two groups, different pathogenic variants or pathovars have been identified (2, 9). The IPEC pathogroup comprises enteropathogenic (EPEC), shiga toxin-producing (STEC), enterohemorrhagic (EHEC), enteroaggregative (EAEC), enterotoxigenic (ETEC), and enteroinvasive (EIEC) *E. coli*, with typical virulence factors used as specific genetic markers for these pathovars (2, 10). ExPEC contains uropathogenic (UPEC), neonatal meningitis (NMEC), sepsis-associated (SEPEC), and avian pathogenic (APEC) *E. coli*. In contrast to IPEC, this subdivision is based on the clinical manifestation and source of isolation of strains, but generally without pathotype-deterministic virulence factors (2). In addition, there are specific instances in which no specific label for the associated ExPEC pathovars exists (e.g., in the case of skin and soft tissue infections or pneumonia). While ExPEC and IPEC pathogroups are typically mutually exclusive, exceptions occur (2). For example, diffuse adhering *E. coli* (DAEC) is associated with persistent diarrhea in children with Crohn’s disease but also causes urinary tract infections (2). Moreover, multiple strains with hybrid pathovars have been described, including combinations of IPEC and ExPEC pathovars. Examples of this are the hybrid EAEC/STEC strain responsible for a deadly outbreak in 2011 in Germany (11) or the EAEC/UPEC hybrid detected in Australian patients with a UTI (12).

To account for the large intra-species diversity, several typing schemes have been developed for *E. coli*. One broadly used scheme divides *E. coli* into phylogenetic groups (PGs) using 17 housekeeping genes (13). In a clinical context, the Achtman multilocus sequence typing scheme, based on the allelic variants of seven housekeeping genes (14), divides *E. coli* strains into sequence types (STs) and has proven to track the typical ExPEC lineages effectively (15). In total, the Achtman scheme currently accounts for more than 10,000 STs, but the majority of ExPEC infections (>85%) are linked to only 20 STs (15), with ST131 being the most represented. While several STs can also be linked to an IPEC pathotype, with ST11 being the most notable one, a serotyping scheme that considers variants of surface antigens (O for lipopolysaccharide and H for flagella) is more broadly used in the IPEC field. The most notorious IPEC serotype is O157:H7 (2, 16, 17), which in the majority of cases corresponds to ST11. While IPEC and their subgroups can be successfully identified and classified based on the profile of their VAGs (2, 10), multiple attempts to define a similar scheme for ExPEC have proven unreliable (2, 15).

*E. coli* infections have an enormous impact on human and animal health, our healthcare system, and our economy (2, 6, 15–18). For example, 73,000 yearly cases of intestinal illnesses linked solely to *E. coli* O157:H7 were reported in the United States between 1982 and 2002, with 350 individual outbreaks and more than 17% of cases requiring hospitalization (17). For ExPEC, the yearly incidence of infections in the United States was around 9 million in 2003, with direct associated costs reaching 2 billion USD (19). As ExPEC lineages have only increased globally since then (15), the current impact is likely even larger. Furthermore, ExPEC are also prominent opportunistic pathogens of other mammals and birds (20), causing significant issues in the animal food-production sector and concerns regarding the One Health approach (6, 21, 22). Similarly, food-producing animals suffer from IPEC-related diseases, and livestock can serve as a reservoir for IPEC strains, with 75% of O157:H7 human outbreaks being traced back to cattle (6, 23). In addition, *E. coli* and especially ExPEC are commonly linked with antibiotic resistance genes (ARGs), which may greatly contribute to treatment failures in human and veterinary medicine and enhance the spread of antimicrobial resistance (AMR) (2, 6). Despite the significant burden that ExPEC and IPEC impose on our society, and despite ExPEC being the number one cause of healthcare-associated infections, bloodstream infections, and sepsis in the United States (8, 24–26), the pathogenic strains of *E. coli* have often been somewhat neglected and have not always received adequate attention (2). This is reflected in tendencies to leave out *E. coli*, at least initially, from important bacterial surveillance schemes such as those of the U.S. Centers for Disease Control (CDC) or the so-called ESKAPE pathogens (2).

At the moment, it remains unclear what determines the pathogenicity and pathogenic efficiency across different *E. coli* strains. For example, it is not well understood how a small proportion of *E. coli* STs, including the notorious ST131 (7, 15), became responsible for the majority of global ExPEC infections. The answers to such questions may lie in large genome collections, such as EnteroBase (EB), which is the most comprehensive MLST-based *E. coli* genome collection (27). Previous studies have already demonstrated the advantages of such genomic analyses. For instance, a collection of more than 10,000 *E. coli* genome sequences has been used to update the division into PGs and identify several new ones (28). Other examples include the investigation of the role of the ColV plasmid in the evolution of ST58, which belongs to the typical ExPEC STs, using 34,364 draft *E. coli* genome assemblies from EB (29), and a characterization of *E. coli* pangenome diversity within and across STs using a curated data set of 20,577 *E. coli* assemblies from EB (4). However, a main limitation of these genome collections remains a lack of sufficient metadata accompanying the sequences, especially in the context of infections. This currently restricts the usability of these large collections in developing an understanding of what makes a strain pathogenic and a pathogen successful.

One factor that could contribute to pathogenicity is the presence of a type VI secretion system (T6SS). The T6SS is a nanomachine that injects effectors into target cells and belongs to the broader family of contractile injection systems. Effector molecules are highly diverse, and target cells can be either bacteria or eukaryotic cells (30–33). As such, the contribution of T6SSs to pathogenicity can be both indirect (by mediating inter-bacterial competition to ensure niche colonization and facilitate nutrient uptake) and direct (by mediating pathogen-host interactions) (34–39). T6SSs are versatile multiprotein complexes, requiring more than 10 core proteins (32), and can be divided into four main classes: T6SS^i^, T6SS^ii^, T6SS^iii^, and T6SS^iv^. Among these, T6SS^i^ stands out as the most prevalent, and it is further divided into six subclasses: T6SS^i1^, T6SS^i2^, T6SS^i3^, T6SS^i4a^, T6SS^i4b^, and T6SS^i5^. Although T6SSs can be found across a wide range of Gram-negative species, including well-known pathogens from the *Pseudomonas*, *Salmonella*, *Campylobacter*, *Vibrio*, *Burkholderia*, *Serratia*, *Edwardsiella*, and *Enterobacter* genera (40–43), not all bacteria have such a secretion apparatus. In fact, differences in prevalence have been reported within the same species, and even among closely related strains (32). In the case of *E. coli*, three T6SS^i^ subclasses of distinct phylogenetic origin have been detected: T6SS^i1^, T6SS^i2^, and T6SS^i4b^. In the *E. coli* field, they are commonly referred to as T6SS1 (corresponding to T6SS^i2^), T6SS2 (T6SS^i1^), and T6SS3 (T6SS^i4b^). In the literature, the associated nomenclature is not unified, and various terms have been used to describe T6SSs and their subdivision. Terms such as types (44), forms (45), subtypes (44), class (30), clusters (44), phylogenetic groups (46), groups (47), locus (47–49), subclass (50), and subgroups (51) are used apparently interchangeably. To ensure consistency within this manuscript, we stick to the terms class (T6SS^i^), subclass (T6SS^i1^, T6SS^i2^, T6SS^i4b^), and groups (referring to further subdivision of subclasses). In *E. coli*, a total of 13 core genes (*tssA–tssM*) are required for T6SS^i^ functionality. These genes can be accompanied by additional genes determining the specificity of the spike (38, 46), by variable (toxic) effectors, and by immunity genes that neutralize effector toxicity for cognate T6SS-harboring cells (32, 46).

In this study, we aimed to assess the prevalence and distribution of different T6SSs in *E. coli* to develop better insight into their potential contribution to pathogenicity in this species. While previous studies have already focused on T6SSs distribution across different PGs of *E. coli*, they represent smaller-scale studies with limited numbers of strains or strains of limited origin (e.g., considering only a subset of STs) (47, 49, 52, 53). To develop a more comprehensive picture, we determined the prevalence and distribution of T6SS subclasses in a large ST-unbiased collection of 131,610 *E. coli* genomes recovered from EB, with updated annotations regarding their clinical properties. For this, we constructed a comprehensive database of *E. coli*-relevant T6SS^i^ regions that combines input from different sources, examined their prevalence across PGs, pathogroups, and pathovars, and determined their co-occurrence with other VAGs and MDR.

## RESULTS

### Composition and annotation of a large-scale *E. coli* genome collection

**Fig 1:**
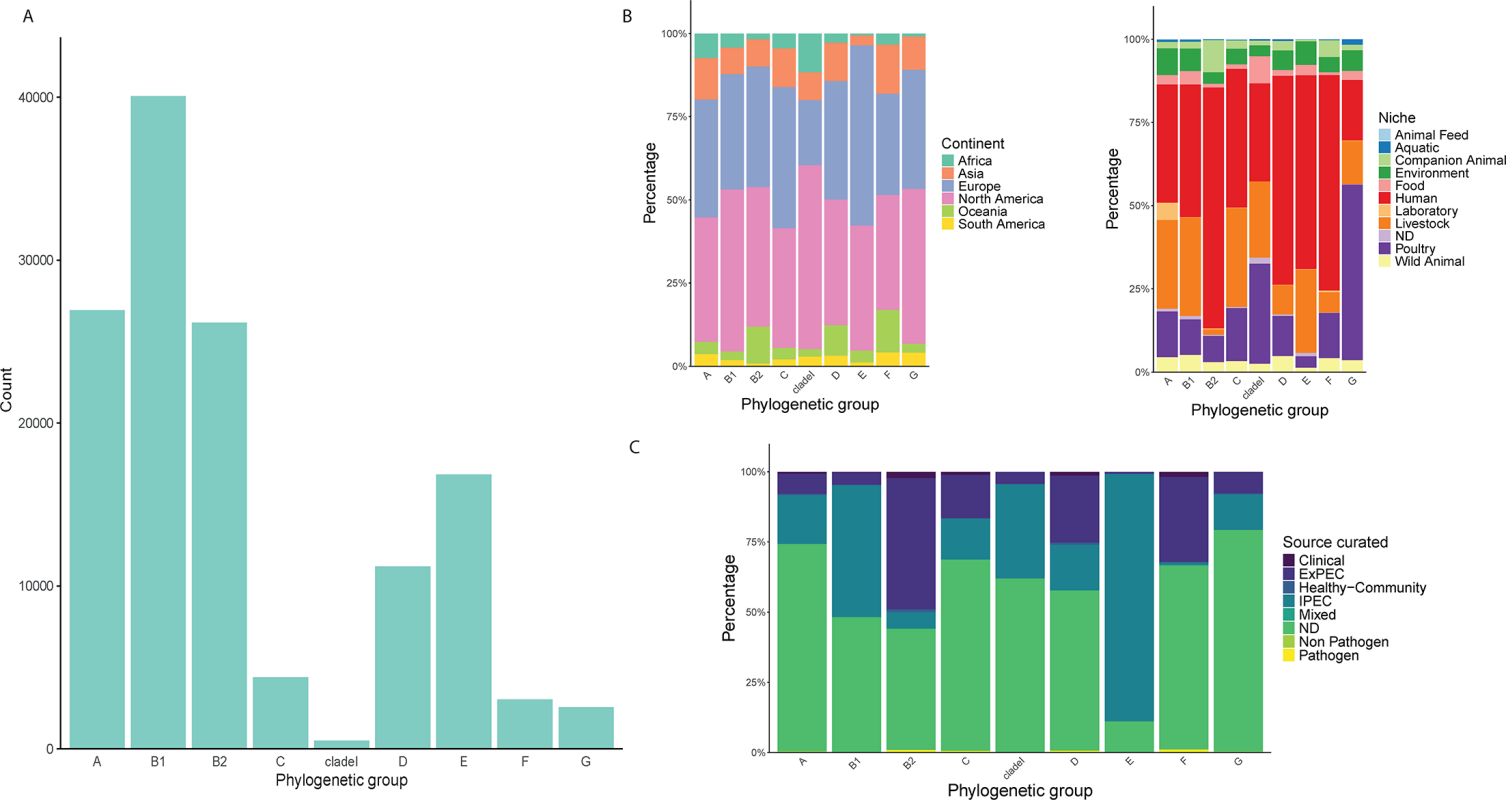
Composition of a large-scale *E. coli* genome collection. (**A**) Bar graph showing the distribution of our collection of genomes (131,610) across major phylogenetic groups. (**B**) Stacked bar graphs showing the relative distribution of selected metadata categories, Continent (left) and Niche (right), within the phylogenetic groups. (**C**) Stacked bar graph showing the relative distribution of metadata category Source curated*,* which reflects the clinical characteristics of genomes.

To uncover T6SS distribution across *E. coli*, we first put together a large-scale *E. coli* genome collection. For this, we downloaded a total of 136,051 available *E. coli* genomes with metadata from EB. We focused on sequences with sufficient metadata and performed additional annotations to extract more clinically relevant information about the strains (see Materials and Methods). We identified PGs using the ClermonTyping tool (54), merged these results with those already present in the EB metadata, and filtered out genomes that showed irregular PG results (see Materials and Methods). After additional filtering (see Materials and Methods), we ended up with a collection of 131,610 genomes. The distribution of these genomes across PGs is shown in Fig. 1A. We found that PG B1 was the most prevalent (40,059 genomes) and clade I the least prevalent (489 genomes) (Fig. 1A; Table 1). Relative distributions within PGs based on the geographical location (Continent) of genomes indicated representation from all continents (except Antarctica), with a roughly similar distribution across PGs and a clear bias toward genomes from Europe and North America (Fig. 1B). A subdivision based on the niche from which strains were isolated (Niche) revealed a general bias in our collection toward genomes coming from humans, although we could still identify notable differences between PGs (Fig. 1B). For example, B2 contains a higher fraction of genomes of human origin than other PGs, but a lower fraction of livestock origin. On the other hand, PG G is enriched with genomes coming from poultry.

**TABLE 1:** Prevalence of T6SS^i^ subclasses (Presence and Completeness) across selected metadata categories^*a*^

Category	N	PT6SSi1	PT6SSi2	PT6SSi4b	P-Mul	CT6SSi1	CT6SSi2	CT6SSi4b	C-Mul
*E. coli*	131,610	55.9%	28.3%	1.3%	14.7%	40.4%	20.7%	1.3%	7.8%
PG									
A	26,922	26.0%	4.8%	2.4%	3.0%	21.7%	3.5%	2.4%	2.3%
B1	40,059	69.5%	22.6%	1.9%	20.4%	53.0%	19.3%	1.8%	15.1%
B2	26,148	38.1%	90.7%	0.0%	36.7%	11.7%	60.3%	0.0%	11.2%
C	4,381	40.6%	3.7%	0.0%	3.4%	38.6%	3.1%	0.0%	2.9%
Clade I	489	44.2%	3.1%	0.0%	2.0%	22.9%	3.1%	0.0%	1.8%
D	11,207	81.7%	2.5%	2.1%	2.5%	64.9%	2.2%	2.1%	2.1%
E	16,829	95.6%	0.8%	0.3%	0.8%	74.8%	0.7%	0.3%	0.4%
F	3,031	42.7%	0.3%	0.0%	0.1%	39.2%	0.1%	0.0%	0.0%
G	2,544	9.1%	98.5%	0.0%	8.4%	8.6%	90.4%	0.0%	7.7%
Niche									
Animal Feed	145	47.6%	9.0%	0.0%	5.5%	38.6%	6.9%	0.0%	4.1%
Aquatic	604	48.3%	24.7%	0.2%	13.7%	32.3%	22.7%	0.2%	4.8%
Companion Animal	4,595	61.8%	58.3%	0.1%	36.5%	29.3%	53.7%	0.1%	11.8%
Environment	8,171	55.0%	18.2%	0.6%	9.5%	41.6%	14.6%	0.6%	5.0%
Food	3,569	62.1%	18.5%	0.1%	9.2%	48.3%	13.0%	0.1%	6.2%
Human	65,700	56.4%	35.7%	2.4%	17.2%	39.1%	23.2%	2.4%	9.4%
Laboratory	1,566	6.2%	5.1%	0.0%	2.0%	4.7%	4.6%	0.0%	0.9%
Livestock	26,615	59.9%	12.9%	0.0%	9.3%	51.4%	11.0%	0.0%	6.8%
ND	965	56.2%	16.6%	0.6%	12.4%	38.7%	14.8%	0.6%	7.7%
Poultry	14,571	47.8%	27.0%	0.0%	12.8%	30.6%	25.1%	0.0%	4.3%
Wild Animal	5,109	58.9%	21.7%	0.2%	13.1%	43.0%	18.9%	0.2%	6.3%
Source curated									
Clinical	1,136	46.0%	54.6%	3.0%	19.0%	29.7%	29.4%	3.0%	8.1%
ExPEC	20,523	50.0%	60.4%	0.3%	24.5%	27.4%	40.1%	0.3%	8.4%
Healthy-Community	571	49.6%	46.1%	0.2%	20.5%	33.6%	38.0%	0.2%	9.5%
IPEC	42,791	71.0%	15.4%	0.1%	13.1%	57.5%	13.3%	0.1%	10.2%
Mixed	159	34.6%	6.9%	0.0%	2.5%	24.5%	4.4%	0.0%	2.5%
ND	65,828	48.2%	25.9%	2.3%	12.6%	33.8%	19.3%	2.3%	6.0%
Non Pathogen	183	41.5%	27.3%	0.5%	7.1%	33.3%	22.4%	0.5%	2.2%
Pathogen	419	45.8%	52.5%	5.0%	15.8%	30.5%	19.6%	5.0%	6.9%
ExPEC type									
APEC	446	50.4%	46,6%	0,2%	20,6%	28,0%	44,8%	0,2%	6,5%
BSI	4,957	40.9%	66.4%	0.3%	18.7%	23.6%	33.6%	0.3%	5.8%
ExPEC	2,524	51.1%	40.9%	0.0%	18.6%	31.5%	28.4%	0.0%	7.0%
No	42,608	71.1%	15.4%	0.1%	13.1%	57.6%	13.3%	0.1%	10.3%
NS	68,322	48.2%	26.7%	2.3%	12.8%	33.7%	19.6%	2.3%	6.0%
Respiratory	407	41.5%	64.1%	0.0%	20.4%	23.1%	33.2%	0.0%	7.6%
UPEC	12,346	53.6%	61.6%	0.3%	28.0%	28.2%	44.7%	0.3%	9.8%
IPEC type									
EHEC	24,298	76.3%	8.3%	0.0%	8.3%	63.7%	8.2%	0.0%	7.9%
EIEC	356	76.4%	1.7%	0.0%	0.6%	49.4%	1.1%	0.0%	0.0%
EPEC	5,176	50.9%	8.7%	0.0%	8.5%	36.1%	8.5%	0.0%	7.7%
ETEC	2,266	51.1%	3.8%	0.0%	3.1%	42.6%	3.4%	0.0%	1.9%
ETEC/EHEC	102	9.8%	0.0%	0.0%	0.0%	6.9%	0.0%	0.0%	0.0%
ETEC/EPEC	46	26.1%	0.0%	0.0%	0.0%	0.0%	0.0%	0.0%	0.0%
ETEC/STEC	557	38.1%	1.3%	0.0%	1.1%	18.1%	1.3%	0.0%	0.4%
ND	501	59.1%	31.3%	0.4%	19.8%	31.7%	27.7%	0.4%	7.0%
No	88,342	48.6%	34.5%	1.8%	15.5%	32.2%	24.3%	1.8%	6.6%
STEC	9,966	75.3%	40.1%	0.6%	30.8%	59.9%	31.6%	0.5%	20.3%

^*a*^ PT6SS^i1–i4b^ corresponds to the Presence of specific T6SS^i^ subclasses, while CT6SS^i1–i4b^ corresponds to Completeness. P/C-Mul corresponds to genomes with multiple T6SS^i^ subclasses. N specifies the number of genomes in the respective categories. The same type of prevalence analysis for both DBs separately is presented in Table S3.

We also subdivided the genomes based on their clinical relevance (Source curated). This subdivision consisted of three main groups: not determined (ND), IPEC, and ExPEC (Fig. 1C). The largest one, ND, comprised about half of our collection (65,828 genomes) and represents genomes for which this additional annotation could not be assigned based on the available metadata. While this group is expected to contain less pathogenic strains than the other two, it cannot be perceived as synonymous with non-pathogenic and likely houses representatives of all possible lifestyles of *E. coli*, from environmental strains to commensals and pathogens (of unknown proportions/severity). The IPEC group consisted of 42,791 genomes, mostly assigned using the VAGs-based scheme (97% of IPEC genomes). We assigned genomes to the ExPEC group (20,523 genomes) mainly based on the source from which they were isolated. We hereby focused on the presence of *E. coli* in areas associated with symptomatic or asymptomatic infections (e.g., blood or urine). Other subgroups related to the clinical relevance of genomes were smaller, ranging from 1,136 genomes identified as Clinical (referring to an unspecific clinical annotation) to 159 genomes identified as Mixed (referring to mixed IPEC-ExPEC pathotype). The other minor groups were represented by Healthy-Community (571 genomes), Pathogen (419 genomes), and Non Pathogen (183 genomes). Examination of this subdivision across PGs revealed an increased fraction of IPEC genomes in PG B1 and E, while an increased fraction of ExPEC genomes was observed in PG B2, F, and D (Fig. 1C).

The ExPEC and IPEC groups were also divided into several subgroups (see Materials and Methods) and further investigated separately (Tables S4 and S5). This analysis revealed an uneven distribution of the subgroups across PGs, especially apparent for IPEC (Fig. S2). EHEC represents the major pathovar of PG E with a small fraction of other pathovars in this PG, while PG B1 contains similar proportions of EHEC and STEC, and a considerable fraction of EPEC (Fig. S2). We also examined which STs are linked with ExPEC (Table S6) or IPEC (Table S7), and assessed their distribution across ExPEC (Table S4) and IPEC (Table S5) types. This analysis confirmed the role of ST131 as the major ExPEC ST, with 24% of ExPEC-labelled strains corresponding to this ST and only APEC not having ST131 as the most common ST (ST117 was the most dominant ST within this ExPEC type). Besides ST131, only one other ST represented more than 5% of ExPEC strains, namely ST73, with 7%. Within IPEC, the dominance of ST11 is apparent as it represents almost one-third (33%) of IPEC genomes. The only other IPEC ST reaching more than 5% is ST21 (9%). Across IPEC pathovars, ST11 mostly dominated EHEC and was the second most prevalent ST for EPEC, after ST10.

### Construction of a comprehensive database of *E. coli* T6SS components reveals the distribution of T6SS^i^ subclasses across *E. coli* genomes

**Fig 2:**
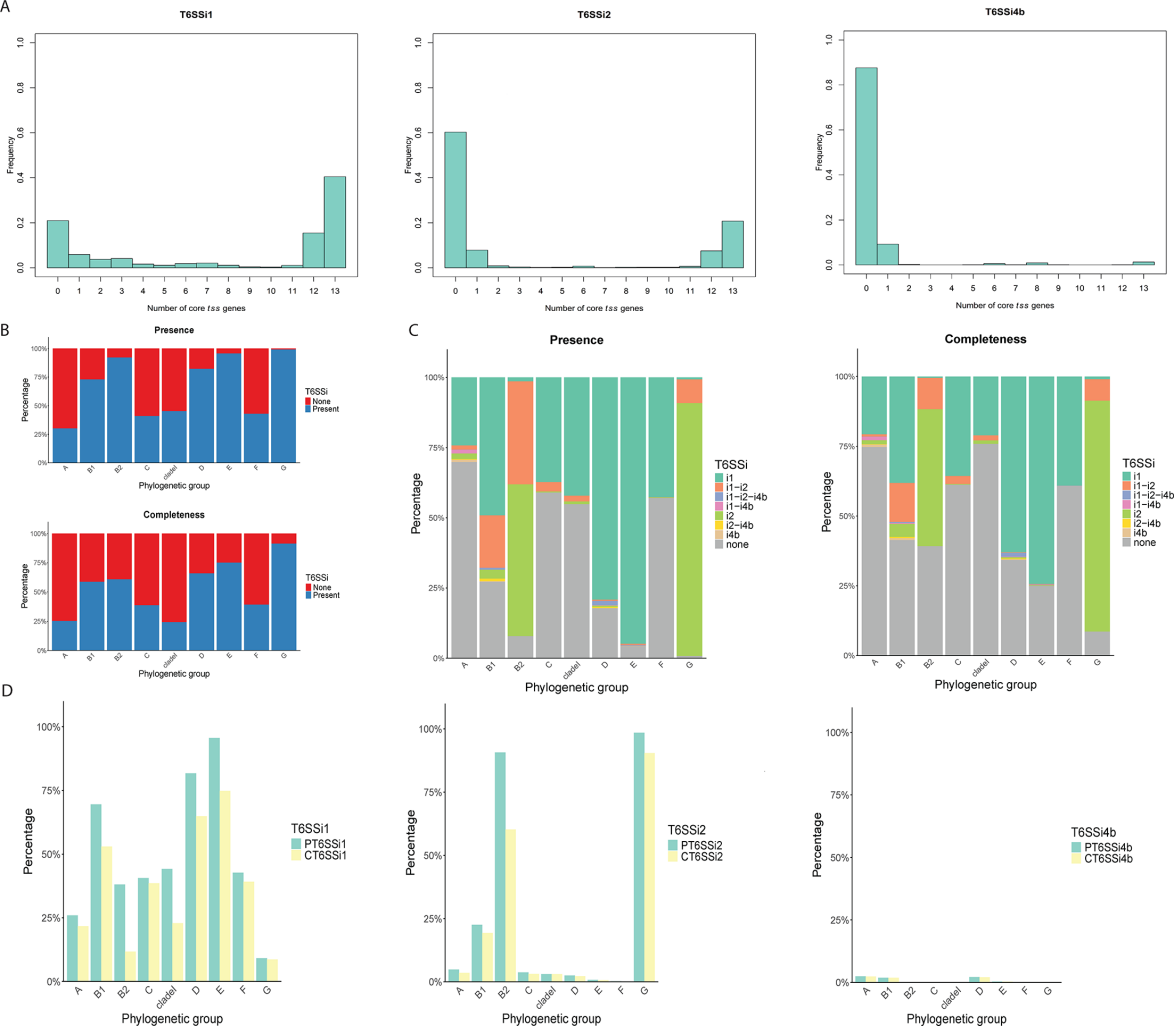
T6SS^i^ subclasses in the collection of genomes. (**A**) Bar graphs showing the fraction of genomes with the indicated number of core *tss* genes (0–13) for T6SS^i1^ (left), T6SS^i2^ (middle), and T6SS^i4^ (right). (**B**) Stacked bar graphs showing the percentage of genomes with at least one subclass of T6SS^i^ detected as Present (top) or Complete (bottom) across the phylogenetic groups. (**C**) Stacked bar graphs showing the specific combinations of T6SS^i^ subclasses that were detected as Present (left) or Complete (right) across the phylogenetic groups. (**D**) Bar graphs showing the Presence (green bars) and Completeness (yellow bars) of T6SS^i1^ (left), T6SS^i2^ (middle), and T6SS^i4^ (right) across the phylogenetic groups.

To investigate the distribution of various T6SS^i^ subclasses across our *E. coli* genome collection, we aimed to construct a comprehensive sequence database of *E. coli* T6SS components. For this, we combined sequences from different available sources (see Materials and Methods) and focused on genes annotated as one of the 13 core genes (*tssA–tssM*). Using this database, we evaluated the presence of these genes for each T6SS^i^ subclass across our *E. coli* genome collection. The frequency distributions of the number of detected *tss* core genes revealed an almost binary pattern for each T6SS^i^ subclass (Fig. 2A), where either (almost) all components of a given T6SS^i^ subclass were present or none. We exploited this distribution pattern to assess both the completeness and presence of the three T6SS^i^ subclasses that occur in *E. coli.* Present/Presence (P) corresponds to the detection of at least 12 core genes from the respective subclass, and Complete/Completeness (C) to the detection of all 13 core genes, where we expect the corresponding T6SS to be functional. We chose to include both, as functionality might still be preserved for some members in the Present group (given that our database, although comprehensive, will never be perfectly complete and some of the core gene components can be found in orphan regions [40, 46]).

Upon examination of the Presence and Completeness of T6SSs across *E. coli* genomes, we found that more than half of the genomes contained at least one T6SS (70.3% and 54.3% for P and C, respectively; Fig. 2B). Moreover, we identified a substantial fraction of genomes containing multiple T6SS^i^ subclasses with 14.7% and 7.8% for P and C, respectively (Fig. 2C). Most of the genomes with multiple T6SS^i^ subclasses combined T6SS^i1^ and T6SS^i2^ (14.0% and 7.2% for P and C), but other combinations also occurred (Fig. 2C). A more detailed investigation of the distribution across PGs revealed distinct distribution patterns between subclasses (Fig. 2D). While T6SS^i1^ was broadly distributed and was prevalent across all PGs, the distribution of both T6SS^i2^ and T6SS^i4b^ appeared more limited, to only a subset of PGs. T6SS^i2^ was found in a relatively large fraction of genomes from PGs B1, B2, and G, while T6SS^i4b^ was not very prevalent and only found in a small fraction of genomes of PGs A, B1, and D. In general, we found a good agreement between the Presence of and Completeness of the T6SS subclasses across PGs, with the largest differences observed in PG B2 for both T6SS^i1^ and T6SS^i2^. As a control, we also performed the same analysis using the separate sequences of T6SS components, based on the database they originated from. This analysis revealed the added value of our merged database, as sequences retrieved from separate databases often only partially recapitulated the global distribution pattern of different T6SS^i^ subclasses (Fig. S3). For example, the T6SS^i1^ was almost exclusively linked to B2 in one of the databases (Fig. S3), which is likely an artefact of focusing only on a subset of sequences, given that this association no longer holds when considering all T6SS^i1^ sequences (Fig. 2D).

**Fig 3:**
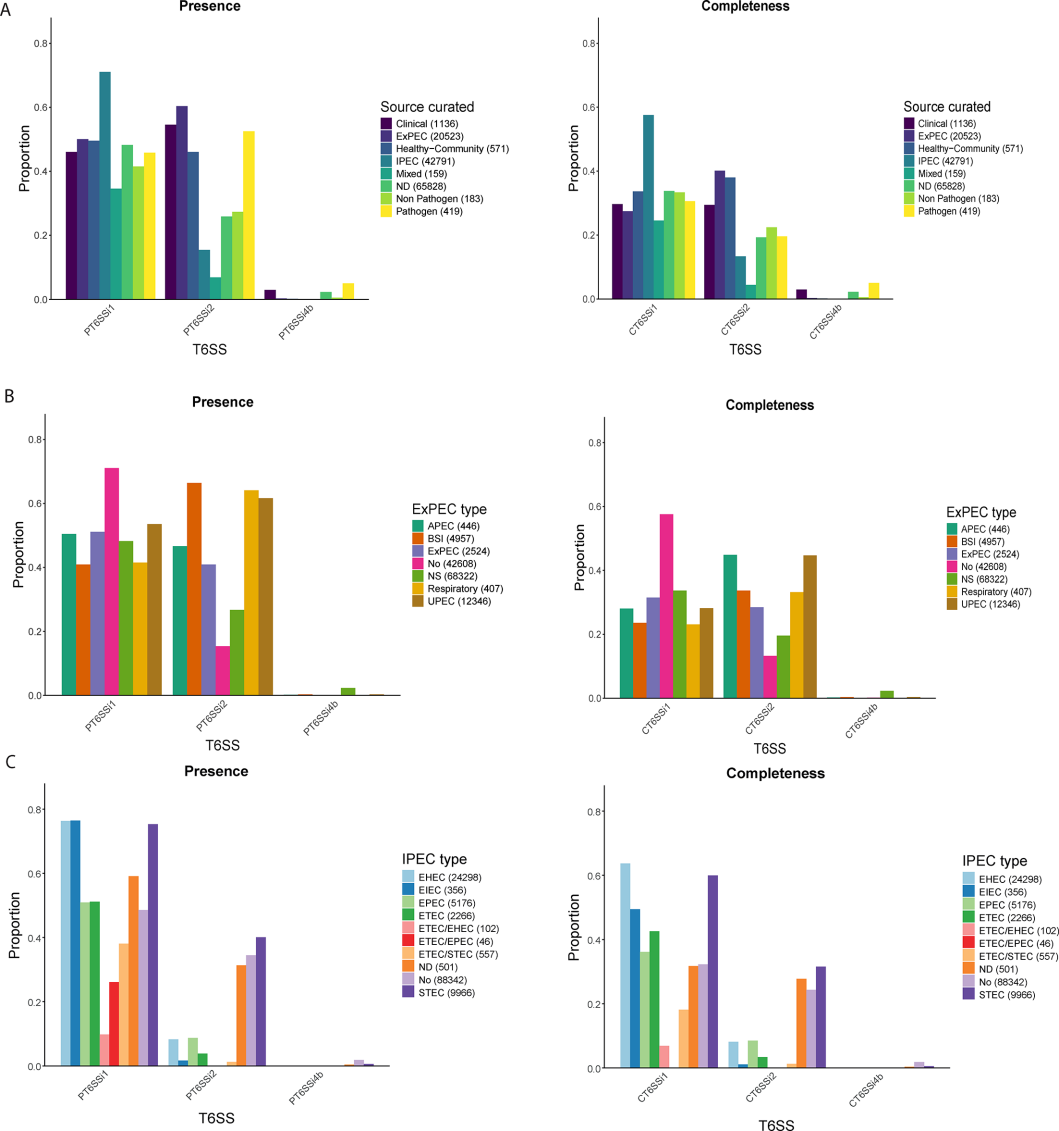
Proportions of T6SS^i^ subclasses in clinically relevant groups. For all panels, the proportion of T6SS^i^ subclasses that were detected as Present is shown on the left, while those that were detected as Complete are shown on the right. (**A**) Bar graphs showing the proportions of T6SS^i^ subclasses across the Source curated category. (**B**) Bar graphs showing the proportions of T6SS^i^ subclasses across ExPEC types. (**C**) Bar graphs showing the proportions of T6SS^i^ subclasses across IPEC types.

To gain further insight into the potential association of T6SSs with the pathogenic properties of strains, we also investigated their distribution across the Source curated category of genomes. This analysis revealed an enrichment of T6SS^i1^ for IPEC genomes (Fig. 3A; Table S8). For T6SS^i2^, the ExPEC genomes were the largest group, although this proportion was statistically indistinguishable from the Healthy-Community group for the Complete T6SS^i2^ (*P*-value < 0.31; Table S8). Within the T6SS^i2^-positive genomes, we observed a strong depletion of IPEC and Mixed genomes in comparison to other groups (Fig. 3A; Table S8). Differences among groups could also be observed for T6SSi^4b^ (Fig. 3A), yet the low absolute values do not allow T6SSi^4b^ to be a relevant marker for any clinical group. In a subsequent step, we explored associations of T6SS^i^ subclasses with ExPEC and IPEC types (Fig. 3B). All ExPEC groups displayed an enrichment in T6SS^i2^ compared to other groups (Table S8), with APEC and UPEC showing the highest association (Fig. 3B). By contrast, for the IPEC group, all major pathovars displayed an enrichment in T6SS^i1^ with the comparative group (No; Table S8), with the exception of the minor hybrid groups (ETEC/EHEC, ETEC/EPEC, and ETEC/STEC). EHEC and STEC contained the largest fraction of genomes with T6SS^i1^ (Fig. 3C), while STEC showed an, for IPEC, atypical association with T6SS^i2^ (Fig. 3C; Table S8).

To explore whether differences in regulatory control exist, we also examined the pattern of experimentally validated T6SS^i^-associated regulatory genes across the Source curated category. We found notable differences for the prevalence of five such genes across these categories (i.e., REG0073, REG0250, REG0251, REG0270, REG0271; Table S10). However, REG0073 (linked with ST11 and ST32, mostly IPEC genomes) and REG0250 (most prevalent and more associated with ExPEC) differed only in two silent mutations (meaning their translation would lead to the same protein). As a consequence, no true difference in prevalence exists as the gene is present in almost all genomes (in one of its two variants). A similar situation applied for REG0251 (linked mostly with IPEC), REG0270 (linked with ExPEC), and REG0271 (somewhat more common in IPEC). REG0251 and REG0270 differ from each other in five silent mutations, and both differ from REG0271 in several other silent mutations. The combined prevalence of these three gene variants (REG0251, REG0270, and REG0271) again reached almost 100%, meaning no true difference in prevalence between genomes exists. In a final step, we also examined correlations between these genes and T6SS^i^ subclasses Presence or Completeness, which did not yield any strong positive or negative associations (Table S11).

### Distribution of T6SS^i^ subclasses across major *E. coli* STs

**Fig 4:**
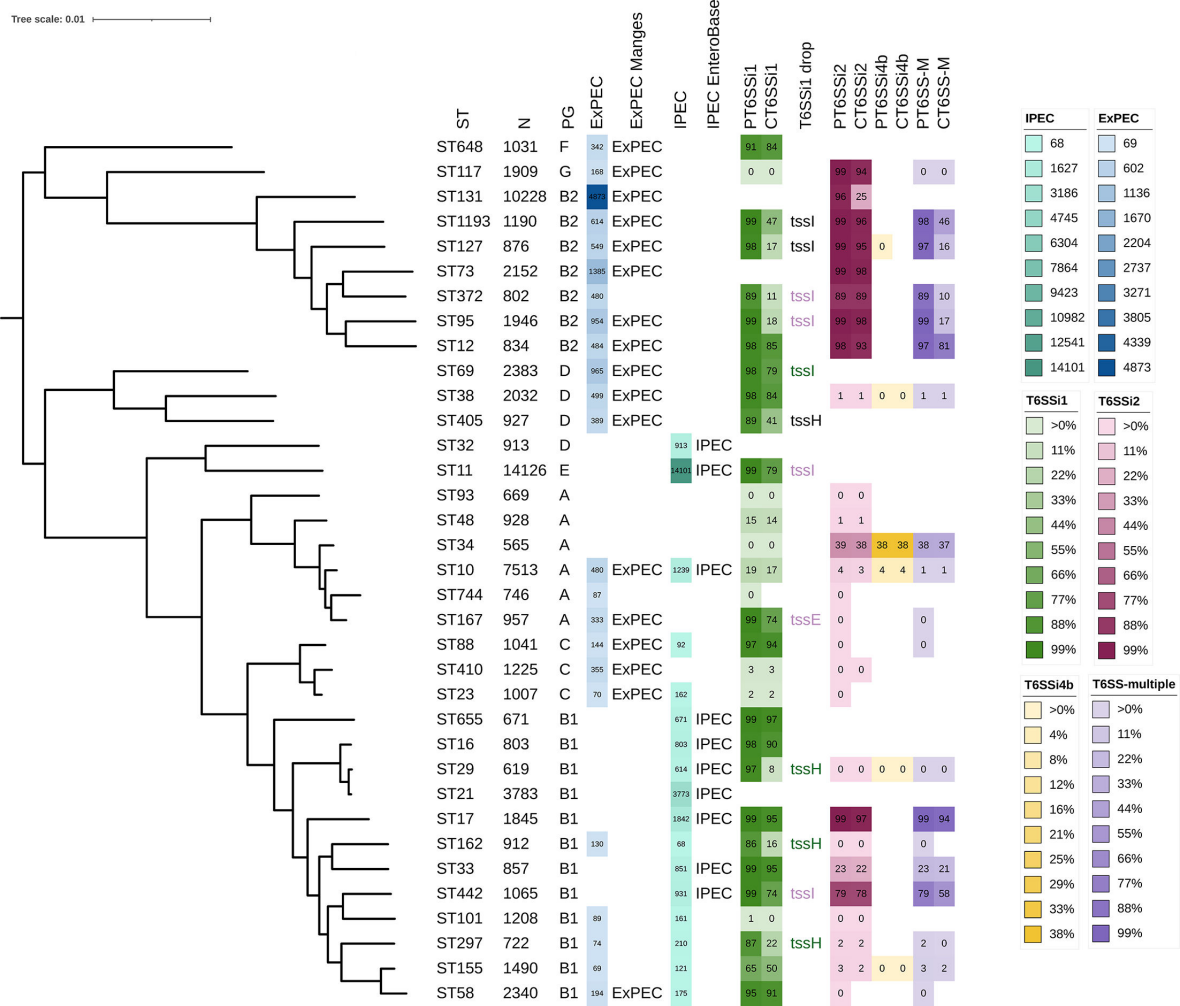
Phylogenetic analysis of the 35 most dominant STs. The metadata included in our phylogenetic tree are sequence types (ST), number of genomes of the respective ST in our collection (N), phylogenetic group (PG), heatmap highlighting the dominant ExPEC STs in our study (threshold of min. 50 ExPEC genomes) (EXPEC), ExPEC label given to 20 dominant ExPEC STs undertaken from reference 15 (ExPEC Manges), heatmap highlighting the dominant IPEC STs (threshold of min. 50 IPEC genomes) (IPEC), IPEC label given to STs which belonged among the 20 dominant IPEC STs determined based on EnteroBase information (IPEC EnteroBase), heatmap showing the prevalence (%) of T6SS^i1^ for each ST (PT6SSi1, CT6SSi1), T6SS^i1^-associated *tss* genes that could be linked to the drop between P and C (T6SSi1 drop) – the genes in black were not found, the genes in purple were found truncated, and the genes in green were identified after additional examination of the T6SS contigs –, heatmap showing the prevalence of T6SS^i2^ for each ST (PT6SSi2, CT6SSi2), heatmap showing the prevalence of T6SS^i4b^ for each ST (PT6SSi4b, CT6SSi4b), heatmap showing the prevalence of multiple T6SS^i^ subclasses for each ST (PT6SS-M, CT6SS-M). P/C corresponds to Present (detection of at least 12 *tss* genes for the specific T6SS^i^ subclass) and Complete (detection of all 13 core *tss* genes for the specific T6SS^i^ subclass), respectively.

In a subsequent step, we examined the distribution of T6SS^i^ subclasses across the 35 most prevalent STs of *E. coli* (each containing >500 genomes). An overview of this analysis is presented in Fig. 4. In general, T6SS^i^ subclasses displayed a more delineated distribution pattern, with stronger enrichments and absences within STs than within PGs. In the majority of cases, a given T6SS^i^ subclass was either present or not within an ST (Fig. 4; Table 2). With the exception of ST155, T6SS^i1^ is either Present (>85%) or almost completely absent (<20%) for specific STs. This absence is not linked to specific PGs, as T6SS^i1^ is not present in ST131 and ST73 (B2), ST32 (D), ST744 (A), or ST21 (B1). ST21 is particularly relevant, given that it represents the second most dominant IPEC ST, yet shows no presence of any T6SS^i^. T6SS^i2^ was present in all STs in PG B2 (ST131, ST1193, ST127, ST73, ST372, ST95, and ST12) and the only ST from G (ST117), which all belong to prominent ExPEC. At the same time, T6SS^i2^ was also prevalent in two STs from the B1 PG linked to an IPEC pathotype, namely ST17 and ST442. We only found T6SS^i4b^ in a fraction of ST34 genomes, where they typically co-occurred with T6SS^i2^. Strains belonging to several other STs, including ST12, ST1193, ST17, and ST442, also contained multiple T6SSs, which typically combined T6SS^i1^ and T6SS^i2^ (Fig. 4).

**TABLE 2:** Prevalence of T6SS^i^ subclasses (Presence and Completeness) in the 35 dominant STs^*a*^

ST	N	PT6SSi1	PT6SSi2	PT6SSi4b	P-Mul	CT6SSi1	CT6SSi2	CT6SSi4b	C-Mul
ST11	14,126	99.4%	0.0%	0.0%	0.0%	79.5%	0.0%	0.0%	0.0%
ST131	10,228	0.0%	96.3%	0.0%	0.0%	0.0%	25.0%	0.0%	0.0%
ST10	7,513	19.9%	4.8%	4.0%	1.8%	17.9%	3.2%	4.0%	1.6%
ST21	3,783	0.0%	0.0%	0.0%	0.0%	0.0%	0.0%	0.0%	0.0%
ST69	2,383	98.9%	0.0%	0.0%	0.0%	79.9%	0.0%	0.0%	0.0%
ST58	2,340	95.3%	0.6%	0.0%	0.6%	91.7%	0.0%	0.0%	0.0%
ST73	2,152	0.0%	99.3%	0.0%	0.0%	0.0%	98.3%	0.0%	0.0%
ST38	2,032	98.7%	1.7%	0.6%	1.7%	84.9%	1.3%	0.6%	1.3%
ST95	1,946	99.5%	99.8%	0.0%	99.4%	18.0%	98.7%	0.0%	17.8%
ST117	1,909	0.1%	99.6%	0.0%	0.1%	0.1%	94.0%	0.0%	0.1%
ST17	1,845	99.3%	99.2%	0.0%	99.1%	95.6%	97.9%	0.0%	94.5%
ST155	1,490	65.7%	3.0%	0.3%	3.2%	50.1%	2.3%	0.3%	2.6%
ST410	1,225	3.9%	0.1%	0.0%	0.0%	3.8%	0.1%	0.0%	0.0%
ST101	1,208	1.9%	0.2%	0.0%	0.0%	0.8%	0.1%	0.0%	0.0%
ST1193	1,190	99.0%	99.3%	0.0%	98.8%	47.2%	96.1%	0.0%	46.6%
ST442	1,065	99.1%	79.5%	0.0%	79.2%	74.1%	78.2%	0.0%	58.0%
ST88	1,041	97.9%	0.9%	0.0%	0.9%	94.4%	0.0%	0.0%	0.0%
ST648	1,031	91.5%	0.0%	0.0%	0.0%	84.2%	0.0%	0.0%	0.0%
ST23	1,007	2.9%	0.4%	0.0%	0.0%	2.7%	0.0%	0.0%	0.0%
ST167	957	99.2%	0.1%	0.0%	0.1%	74.5%	0.0%	0.0%	0.0%
ST48	928	15.2%	1.0%	0.0%	0.0%	14.4%	1.0%	0.0%	0.0%
ST405	927	89.4%	0.0%	0.0%	0.0%	41.9%	0.0%	0.0%	0.0%
ST32	913	0.0%	0.0%	0.0%	0.0%	0.0%	0.0%	0.0%	0.0%
ST162	912	86.6%	0.5%	0.0%	0.3%	16.2%	0.2%	0.0%	0.0%
ST127	876	98.1%	99.3%	0.1%	97.4%	17.1%	95.9%	0.0%	16.3%
ST33	857	99.1%	23.7%	0.0%	23.7%	95.4%	22.6%	0.0%	21.7%
ST12	834	98.4%	98.1%	0.0%	97.0%	85.1%	93.9%	0.0%	81.2%
ST16	803	98.8%	0.0%	0.0%	0.0%	90.3%	0.0%	0.0%	0.0%
ST372	802	89.7%	89.9%	0.0%	89.2%	11.0%	89.2%	0.0%	10.2%
ST744	746	0.1%	0.1%	0.0%	0.0%	0.0%	0.0%	0.0%	0.0%
ST297	722	87.8%	2.8%	0.0%	2.6%	22.0%	2.2%	0.0%	0.7%
ST655	671	99.9%	0.0%	0,0%	0,0%	97,8%	0,0%	0.0%	0.0%
ST93	669	0.1%	0.1%	0.0%	0.0%	0.1%	0.1%	0.0%	0.0%
ST29	619	97.1%	0.6%	0.2%	0.8%	80.0%	0.6%	0.2%	0.8%
ST34	565	0.5%	39.5%	38.9%	38.8%	0.2%	38.8%	38.6%	37.9%

^*a*^ The 35 STs were selected from our data set based on being represented by more than 500 genomes. PT6SS^i1–i4b^ correspond to the Presence of a specific T6SS^i^ subclass, while CT6SS^i1–i4b^ correspond to Completeness, P/C-Mul considers genomes with multiple T6SS^i^ subclasses, and N specifies the number of genomes in the respective category.

As before, we found a good concordance between the Presence and Completeness of T6SS^i^ subclasses, with some notable exceptions (Fig. 4). While T6SS^i2^ is present in the majority of ST131 genomes, only 25% contain a complete T6SS^i2^. This drop has been described in a previous study in which it was linked to an interruption of the *tssM* gene, encoding part of the T6SS membrane complex, by IS*Ec12* (53), a mobile genetic element. For T6SS^i1^, we could identify several STs that displayed large discrepancies (≥15%) between the Presence and Completeness of this subclass. With the exception of ST155, the drop from P to C could typically be attributed to a single *tss* gene per ST (Fig. 4). Inspection of the relevant region revealed three possible scenarios: (i) the missing component was present and intact in the region (ST69, ST29, ST162, and ST297), but its sequence was not present in our T6SS component database (a likely consequence of our database only including experimentally validated T6SSs), (ii) the missing gene was not identified in the contigs carrying the *tss* genes (ST1193, ST127, and ST405), suggesting it was lost, part of an orphan region not included in the T6SS databases, or a bioinformatically predicted T6SS gene located on a different contig, or (iii) the missing gene was truncated (ST372, ST95, ST11, ST167, and ST442) (Fig. 4; Fig. S8). For example, a residue of the IS*3* family transposase was recovered next to the *tssE* of ST167, likely playing a role in its truncation in this particular case.

To highlight the potential clinical relevance of the 35 dominant STs, we annotated them with an ExPEC/IPEC label and indicated the number of genomes within the ST that we assigned to the ExPEC or IPEC category (Fig. 4). The ExPEC label was awarded based on a previous meta-analysis of more than 100 ExPEC studies to determine the most dominant ExPEC STs (15). In general, our own determinations of the number of ExPEC genomes within an ST largely corresponded to these labels, with some notable exceptions. For example, we identified elevated fractions of ExPEC strains in several STs that do not belong to the dominant 20 STs responsible for the large majority of ExPEC infections. This included ST372, but also ST744, ST162, ST101, and ST297. For IPEC, we awarded the IPEC label to STs that belonged to the 20 most dominant IPEC STs determined by our analysis, based largely on the virulence scheme present in EB (27). Similar to ExPEC, the fraction of IPEC genomes within an ST largely corresponded to these labels, with ST23, ST101, ST297, ST155, and ST58 as exceptions. Only specific STs displayed elevated fractions of both ExPEC and IPEC genomes, with ST10 being the most notable example. Further analysis revealed that this observation typically stems from two distinct proportions of strains within an ST (exclusively ExPEC and exclusively IPEC), rather than from a hybrid pathotype. Yet this is not conclusive as a large proportion of the genomes is labelled as ND (Fig. S9).

### T6SS-related traits of *E. coli* ST131 and ST11

**Fig 5:**
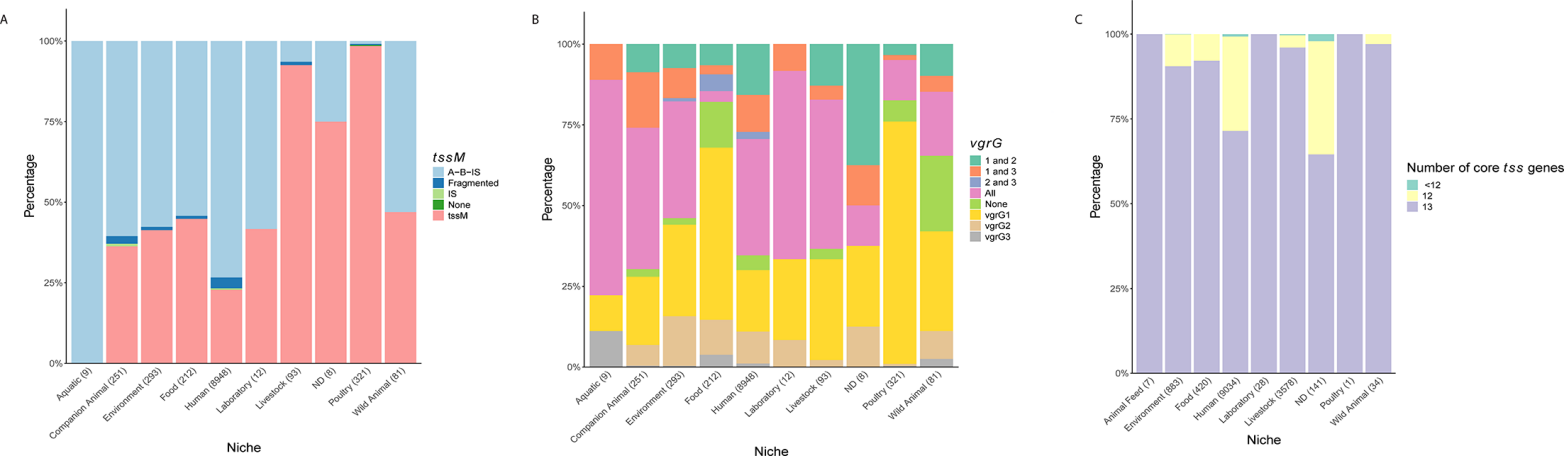
Niche-related T6SS patterns for ST131 and ST11. Calculated *P*-values to highlight which Niche groups differ significantly in their Completeness of T6SS^i2^ (for ST131) or T6SS^i1^ (for ST11) are listed in Table S8. (**A**) Stacked bar graphs showing the relative distribution of T6SS^i2^-related *tssM* or its fragments in different niches. A-B-IS corresponds to the described fragmentation by IS*Ec12* (53) and detection of both fragments (**A, B**) and the IS. Fragmented corresponds to detection of other combinations of fragments (only fragment A or B, both fragments without IS, fragment A and IS, or fragment B and IS), IS to detection of only the IS, None to no detection of any of the elements, and *tssM* to the detection of an intact *tssM* gene. To provide a broader context of *tssM* fragmentation, the same figure for the whole collection of genomes is shown in Fig. S10, which also gives an overview across phylogenetic groups. (**B**) Stacked bar graphs showing the relative distributions of ST131-relevant *vgrG* (here referred to as 1–3) in different niches for ST131. The same figure for the whole collection of genomes is provided in Fig. S10, which also provides an overview across phylogenetic groups. (**C**) Stacked bar graphs showing the Completeness of T6SS^i1^ in ST11 in relation to Niche.

After mapping the general distribution pattern of T6SSs in *E. coli*, we set out to investigate T6SS-related properties of important pathogenic strains in more detail. For ExPEC, we focused on ST131, the major ExPEC ST. This ST also contained a unique pattern of reduced T6SS^i2^ Completeness (Fig. 4), which was previously traced back to a *tssM* fragmentation (53). To examine the prevalence of this fragmentation across ST131, we evaluated the presence of ST131-specific *tssM* fragments (A, B) and IS*Ec12*. This analysis revealed that either the intact *tssM* sequence was present (likely generating a functional T6SS) or both *tssM* fragments and the IS*Ec12* were present (likely generating a non-functional T6SS) (Fig. 5A). We only found other options in very rare instances (Fig. 5A). Fragmentation of *tssM* in ST131 did display a distinct niche-related pattern (Fig. 5A). While some categories were limited in terms of representation and could not reliably be compared to others (i.e, Aquatic or Laboratory), we found a significant difference between Humans and Poultry (*P*-value < 10^−209^) or Humans and Livestock (*P*-value < 10^−61^). Whereas *tssM* was largely fragmented in Humans, it was mostly intact in genomes belonging to the Poultry or Livestock category. Besides this potential *tssM* fragmentation, this region also contained three *vgrG* (*tssI*) genes (53). VgrG encodes a spike protein that, together with the PAAR adaptor protein, forms a membrane-penetrating needle (38, 55), where different VgrG and PAAR variants typically associate with different types of effector molecules (31). We evaluated the presence of these three *vgrG* variants and found a large variability in their distribution across ST131 genomes (Fig. 5B). While different combinations occurred in variable proportions across niches, both Poultry and Food displayed a pattern that deviated from the rest, containing an enrichment of VgrG1 by itself (Fig. 5B).

In parallel, we examined the T6SSs of *E. coli* ST11, the most dominant IPEC and the most represented ST in our collection (14,126 genomes). For this ST, we observed a drop in T6SS^i1^ Completeness (Fig. 4), which was linked to the disappearance of *vgrG*. This disappearance also displayed a niche-related pattern, occurring more in strains from the Human category than in Livestock (*P*-value < 10^−200^; Fig. 5C), the other dominant category. However, given that *vgrG* is a versatile T6SS component that is often located in an orphan region, it is less likely that this drop indeed corresponds to a loss of T6SS functionality. The drop could thus simply reflect the presence of an alternative VgrG variant, not present in our T6SS database, which occurs more in genomes from a Human source.

### T6SS co-occurrence analysis with virulence-associated genes and multi-drug resistance

**Fig 6:**
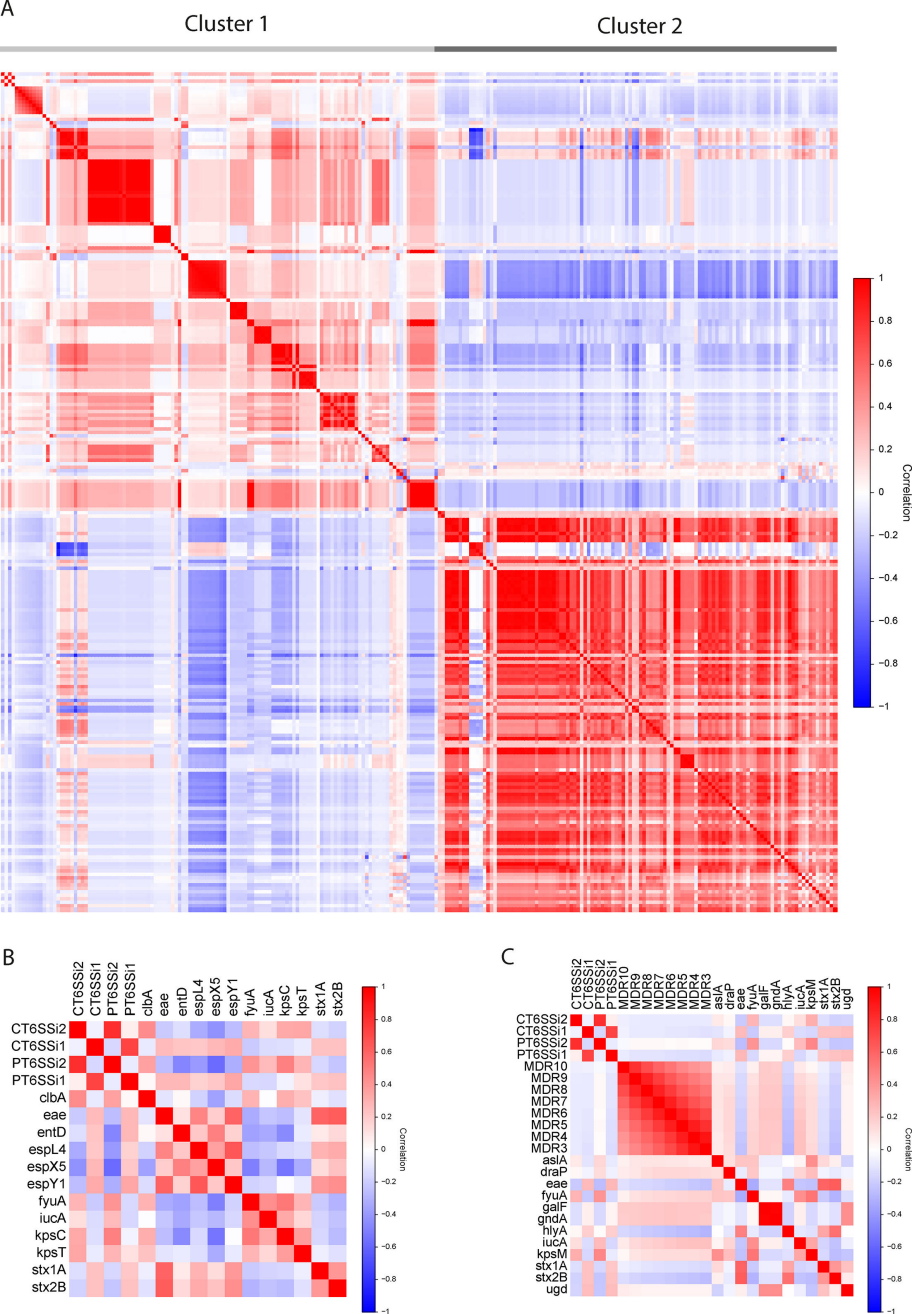
Correlations between T6SS^i^, VAGs, and MDR. (**A**) Clustergram depicting the correlation coefficients between the Presence and Completeness of T6SS^i^ subclasses, a collection of 227 VAGs recovered from VFDB, and multi-drug resistance. Cluster 1 contains typical ExPEC VAGs, while cluster 2 contains typical IPEC VAGs. Figure S11 provides a zoom in on the separate clusters. (**B**) Heatmap highlighting the subset of individual VAGs displaying strong positive and negative correlations between Presence and Completeness of T6SS^i^ subclasses. (**C**) Heatmap highlighting correlations between VAGs or T6SS^i^ subclasses and MDR.

In a final step, we sought to investigate the co-occurrence of T6SSs with other VAGs and ARGs, given that links between these and T6SSs have been described before (53, 56) and could be important in a pathogenic context. For this, we examined the correlation between the Presence and Completeness of T6SS subclasses, VAGs, and multi-drug resistance (MDR; in this case determined by specified amounts of ARGs). The results of this analysis are summarized in the clustergram of Fig. 6 and Table S9 and revealed two dominant clusters within the *E. coli* VAGs, which largely corresponded to IPEC and ExPEC-associated virulence factors. In line with our previous results based on the clinical associations and phylogeny of genomes (Fig. 2 and 3), T6SS^i1^ clustered with IPEC VAGs, while T6SS^i2^ clustered with ExPEC VAGs (Fig. 6A). Within the ExPEC-associated VAGs, we could identify several smaller subclusters that displayed strong correlations, indicating that these sets of VAGs typically co-occur (Fig. 6A, Cluster 1). We did not observe such smaller clusters for IPEC-associated VAGs, which mostly all co-occurred, with the exception of colonization factor antigen I represented by *cfaA* (Fig. 6A, Cluster 2). In general, we found that most VAGs displayed mild positive correlations with one T6SS^i^ subclass and negative for the other, with the low prevalence of T6SS^i4^ precluding a reliable analysis for this subclass.

While no correlations between the Presence and Completeness of different T6SSs and individual VAGs were extreme (Fig. 6; Table S9), we did find some notable positive and negative ones (Fig. 6B). The highest positive correlation for T6SS^i1^ was with *espY1*, which is linked to Type III secretion system (T3SS) effectors (57, 57). T6SS^i1^ correlated positively with enterobactin (*entD*) (58) and shiga-like toxins (*stx2B*, *stx1A*, *eae)* (2), and negatively with siderophores, including yersiniabactin and aerobactin *iucA*. On the other hand, T6SS^i2^ showed a negative correlation to other types of T3SS effectors—*espL4* and *espX5* (57) and was positively correlated with yersiniabactin represented by *fyuA* (59) and colibactin (*clbA*). T6SS^i2^ was also correlated positively with capsule-related genes such as *kpsC* and *kpsT* (60).

For MDR, we found a mild negative correlation with Complete T6SS^i1^ and T6SS^i2^ (Fig. 6C), for all levels of MDR definition (eight groups, ranging from minimally three to minimally 10 ARGs; see also Materials and Methods). In general, MDR also appeared to co-occur more with ExPEC-associated VAGs than with IPEC ones. Mild positive correlations with MDR were observed mostly for genes related to the capsule, such as *kpsM*, *galF*, *gndA* or *ugd* (60, 61), yersiniabactin, aerobactin, adhesion (*draP*), or invasion (*aslA*). Mild negative correlations were observed for IPEC-associated shiga-like toxins. Interestingly, hemolysin A (*hlyA*) correlated mildly but most negatively from all the VAGs with MDR. This exotoxin is considered one of the typical ExPEC-associated markers (2) and has been proposed as a high-priority target for ExPEC vaccine development (62). However, our analysis showed that while it can be detected prevalently in some dominant ExPEC STs (ST73, ST127, ST372, and ST12), it is more often associated with dominant IPEC STs (ST11, ST17, ST21, ST32, ST33, ST16, ST29, ST442, and ST655) (Fig. S1C; Table 1) and overall, it is present in 22% of ExPEC and 71% of IPEC.

### VAG-based clustering of T6SS-positive genomes reflects their clinical relevance, T6SS^i^ subclass, and niche

**Fig 7:**
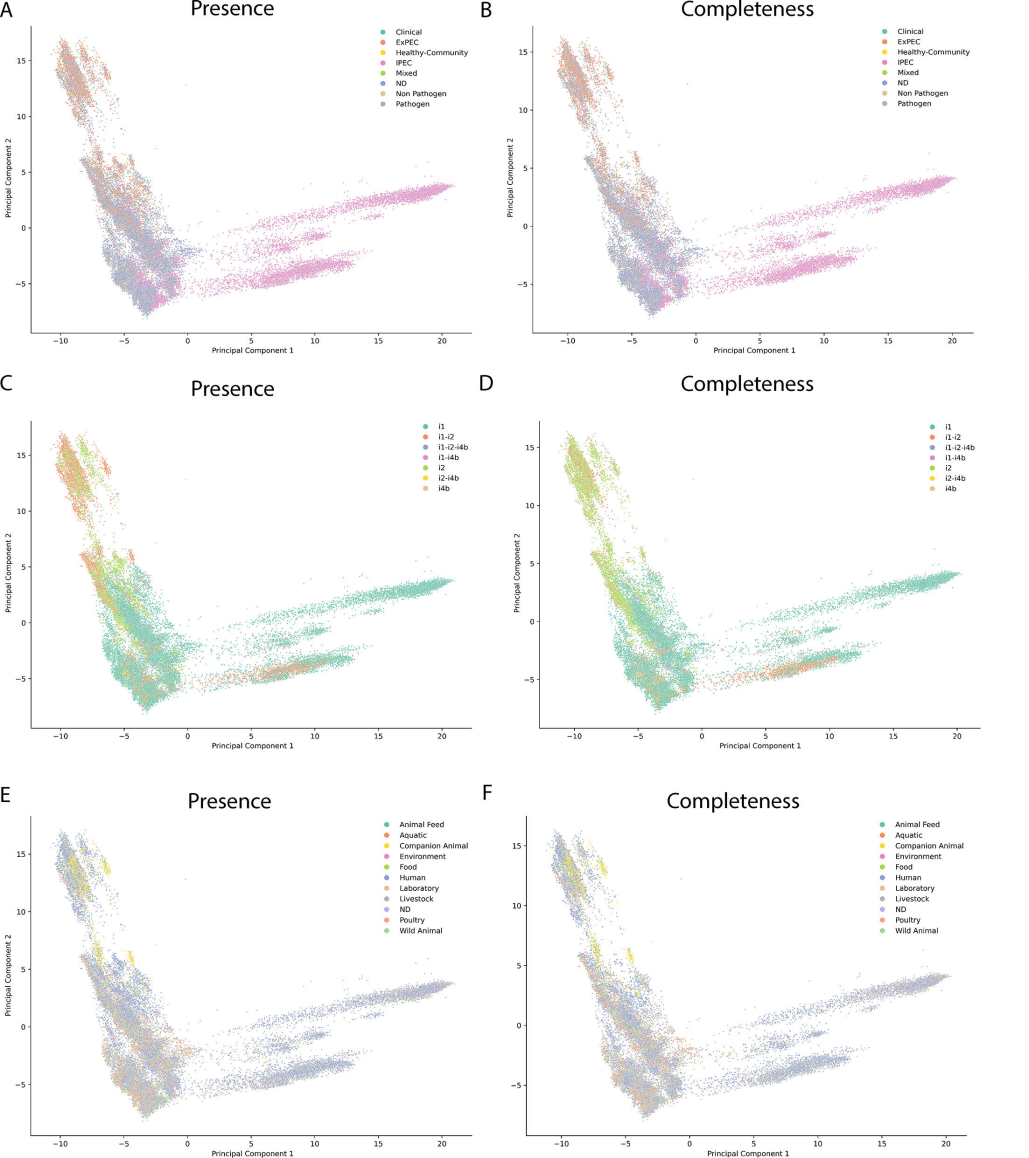
PCA based on VAGs for T6SS^i^-positive genomes. (**A**) Scatterplot displaying the PCA decomposition for Source curated based on Presence (detection of at least 12 *tss* genes for the specific T6SS^i^ subclass). (**B**) Scatterplot displaying the PCA decomposition for Source curated based on Completeness (detection of all 13 core *tss* genes for the specific T6SS^i^ subclass). (**C**) Scatterplot displaying the PCA decomposition for T6SS^i^ subclasses or their combinations based on Presence. (**D**) Scatterplot displaying the PCA decomposition for T6SS^i^ subclasses or their combinations based on Completeness. (**E**) Scatterplot displaying the PCA decomposition for Niche based on T6SS Presence. (**F**) Scatterplot displaying the PCA decomposition for Niche based on T6SS Completeness.

Given that the presence of different VAGs could be linked to the presence of different T6SS^i^ subclasses, to different clinical relevance (IPEC vs. ExPEC), and to different environmental niches, we aimed to provide a more general overview of potential associations among T6SS^i^-positive genomes. For this, we performed a principal component analysis (PCA) using the presence/absence of all potential VAGs as input. The outcome of this PCA is shown in Fig. 7, in which each dot represents an individual T6SS^i^-positive genome (for both Presence and Completeness), colored according to its Source curated (Fig. 7A and B), T6SS^i^ subclass (Fig. 7C and D), and Niche (Fig. 7E and F). While these plots only provide a visual representation of associations between different T6SS^i^-positive genomes, some clear trends already emerge. First, a clear differentiation between ExPEC and IPEC strains becomes apparent, with genomes belonging to other Source curated categories distributed among them, albeit mostly among ExPEC (Fig. 7A and B). Similar as before, IPEC strains were mostly associated with T6SS^i1^, although one large subcluster contained strains with both a T6SS^i1^ and a T6SS^i2^ (Fig. 7C and D). Almost all IPEC strains were isolated from human sources (Fig. 7E and F). ExPEC strains, on the other hand, were clearly a more heterogeneous group. Different ExPEC clusters, typically intermixed with genomes from other Source curated categories, contained different T6SS subclasses or combinations thereof (Fig. 7C and D) and were isolated from various sources (Fig. 7E and F). Together, this analysis provides a visual representation of the VAGs-based diversity of T6SS-positive genomes and how this VAGs-based clustering of genomes reflects other properties such as their clinical relevance, T6SS^i^ subclass, and niche.

### Prediction of pathogenic groups based on T6SSs and virulence-associated genes

In a final step, we sought to exploit the associations between genomic properties (i.e., presence of T6SSs and VAGs) to predict the pathogenic potential of genomes, and whether these are more ExPEC- or IPEC-like. For this, we first trained eight different models to predict whether a genome belongs to the IPEC or ExPEC group based on its VAGs genes and T6SSs Presence or Completeness. While the performance of all models was very high (Table S12), the extra trees model displayed the highest accuracy. The trends in relative feature importance for this model are presented in Fig. S12. While the specific positions somewhat depend on different iterations of running the model, we observed that both notorious IPEC (such as *stx*) and ExPEC (such as *kps* and *papB*) markers scored high (Fig. S12). We then applied this best-performing model to make predictions for genomes belonging to the ND group (of the Source curated category). This analysis revealed that only a minor part of these genomes was predicted to be IPEC-like (less than 5% of the whole collection), and a majority had genomic similarities to ExPEC (Table S13). These findings are in line with our PCA-based analysis showing ND genomes mostly clustering among the ExPEC genomes (Fig. 7A and B). This result was also not entirely unexpected, given that IPEC strains are typically identified based on a well-defined genomic VAGs scheme. Notably, repeating the same training with a separate category for commensals (using genomes labeled as Healthy-Community or Non-Pathogen as a base) yielded very poor prediction results (Table S14). This subpar performance likely reflects a more general issue with confidently assigning genomes as commensals based on their metadata (see Discussion).

## DISCUSSION

In this work, we compiled a large-scale *E. coli* genome collection to assess the distribution of T6SS subclasses across *E. coli* and investigate their potential association with pathogenicity, virulence factors, and antibiotic resistance. Our analysis highlights both the extensive genomic diversity and the variable distribution of different T6SSs across *E. coli*.

By screening the *E. coli* genomes and their associated metadata, we subdivided the genomes into eight categories based on their clinical relevance/information (Source curated). This division revealed that the majority of ExPEC genomes belonged to phylogroups B2, D, and F (Fig. 1B and 3), which is consistent with previous research (2, 7, 28). Also in line with previous studies (20) was the fact that we found most IPEC genomes in phylogroups B1 and E (Fig. 1B). While the latter PG has the highest relative association with IPEC and contained the most dominant IPEC ST (i.e., ST11, representing approx. one-third of all IPEC strains; Fig. 4), the former contained almost the same absolute amount of IPEC genomes and includes notable IPEC STs such as ST21 and ST17 (Fig. 4). The fourth most dominant IPEC ST, ST10 (a dominant EPEC pathovar), is also commonly detected as a prominent ExPEC ST (15). In our collection, this ST was the third most common (7,513 genomes; Table 2), with only a small proportion linked to IPEC (1,239 genomes) or ExPEC (480 genomes) (Fig. S9). An explanation for this observation could stem from this ST containing 0 ST-specific core genes in its pangenome (4), which indicates a lack of shared accessory genome within this ST in addition to the general *E. coli* core genome. Therefore, it is likely that this generalist lineage, which is often commensal (4), can possess specific accessory genes that enable differentiation into IPEC, ExPEC, or hybrid IPEC/ExPEC pathogroups. While the majority of strains fall into a single category (either IPEC or ExPEC), reports of a hybrid aEPEC/ExPEC pathotype of ST10 do exist (63).

While we largely relied on the established virulence scheme to assign an IPEC label to strains, this was different for the ExPEC label. Given that virulence-based schemes have proven unreliable to identify ExPEC (2, 15), we opted for a metadata-based approach. For the further subdivision into ExPEC pathovars, we used the classical ExPEC labels such as UPEC, BSI (classically named SEPEC), and APEC, but also added a Respiratory group. We added this last category as *E. coli* has the potential to cause respiratory infections, albeit with low incidence (64), which is also confirmed by our analysis (407 out of 20,523 ExPEC genomes). For UPEC- and BSI-related genomes, the most dominant STs were identical (i.e., ST131, followed by ST73, ST69, ST95, and ST1193) (Table S4), which aligns with previous reports of the potential of urinary tract infections to progress into bloodstream infections and cause sepsis (65, 66). Within the 35 most prevalent STs of *E. coli* that we identified (Fig. 4), we generally found a good agreement between our ExPEC assignment and that of a previous study (15). The most notable difference was ST372, which was previously not identified as a dominant ExPEC ST, yet contained 480 ExPEC genomes in our analysis. This discrepancy could be explained by the strong association of this ST with Companion animals, causing it to be overlooked. At the same time, it is important to acknowledge that our metadata-based assignment of categories also has limitations. For example, we found that PG G was not strongly associated with ExPEC, in contrast to previous reports (21). However, this is likely a consequence of its link with avian, and especially poultry, sources, which are less likely to have ExPEC-relevant metadata reported. In addition, the assignment is also not straightforward due to potential secondary contamination of poultry body parts in slaughterhouses. Similarly, we did not find a large representation of the APEC pathotype (446 genomes) across our collection, which likely reflects the same constraints of our approach (rather than APEC truly being a rare pathotype).

For the examination of the distribution of T6SS subclasses across *E. coli*, we made a distinction between Present (i.e., detection of at least 12 core genes) and Complete (i.e., detection of all 13 core genes). While Present can be seen as a liberal approximation of functionality, it likely represents an overestimation as the interruption of a single core gene has been reported before (53) and is something we also observed in our analysis (Fig. 4). Complete, on the other hand, likely represents an underestimation of functionality. This is an inherent consequence of working with databases of T6SS with confirmed functionality, which will never be complete. As a result, we expect the true levels of functional T6SS to lie somewhere between the reported prevalence of Complete and Present. Importantly, as we used a bioinformatics-based approach, the functionality can only be presumed, not confirmed, especially considering the thresholds we used for query coverage and identity (85%). Despite these limitations, our approach enabled us to map the prevalence, distribution, and diversity of T6SSs across *E. coli* at an unprecedented scale.

Our general analysis revealed a peculiar distribution pattern of T6SS^i^ subclasses across *E. coli*, where T6SS^i1^ was broadly distributed across all PGs, T6SS^i2^ was mostly associated with PGs B1, B2, and G, and T6SS^i4b^ was only found in a small fraction of genomes belonging to PGs A, B1, and D (Fig. 2C). The distribution pattern of T6SS^i2^ did not correspond to that of a previous study, which indicated a main association with PG D. However, two main factors could underlie this discrepancy. First, Ma et al. (47) focused on T6SSs solely in APEC and performed their screen on a much smaller scale (472 APEC genomes, of which only 69 contained a T6SS^i2^). Despite this limitation, their findings were taken over and extrapolated across *E. coli* in reviews and research articles concerning T6SS in *E. coli* (38, 46, 48). As such, our findings reveal the importance of performing large-scale studies before extrapolating certain findings to a larger group of strains. Second, the PG assignment has evolved over time. For example, ST117 is one of the most prevalent APEC STs (21, 67) (also the most prevalent in this study) and is currently assigned to the relatively recent PG G, in which it represents the vast majority of strains (54). However, in 2013, the assignment, using the previous scheme (68), was PG D. Therefore, it is possible that the evolving PG scheme underlies at least part of the observed discrepancies. As such, we believe it is crucial to always confirm the correct PG classification, especially given that incorrect assignments tend to persist over time and lead to confusion (e.g., ST117 can still be found reported as PG D (69) or as PG F (53) in the recent literature).

Closer examination of T6SSs in ST131, the most notable ExPEC ST, revealed a number of potentially relevant properties in terms of its reduced Completeness (Fig. 5A). While this was due to a *tssM* fragmentation in ST131, which had been reported before (53), we also observed a clear niche-related pattern of this fragmentation (Fig. 5A). The disruption was most prevalent in Humans, yet almost absent in Poultry and Livestock. While this observation challenges the notion that ExPEC clones can be easily exchanged among these niches, it aligns with another study that showed that avian ST131 formed a distant cluster with specific VAGs (70). At the same time, this truncation could provide valuable information on the dominance of ST131 within ExPEC infections, given that most other ExPEC STs from the B2 phylogroup still contained a Complete T6SS^i2^ (Fig. 4). This observation suggests that T6SS^i2^ potentially contributes to the initial evolution of successful ExPEC lineages, and that its subsequent loss of functionality confers additional cellular properties relevant for the pathogenicity of ST131. These properties currently remain speculative but could include enhanced fitness and stress resistance (as reported for *Campylobacter jejuni* in the presence of bile salts [71]), or enhanced horizontal gene transfer. Horizontal gene transfer is known to allow *E. coli to* adapt to new niches, and the large accessory genome of ST131, consisting of over 22,500 genes (72), suggests this is at least one of the factors ST131 has benefited from. In addition, the propagation of conjugation has been linked to the repression of T6SSs (73), and ST131 without functional T6SS has also been linked to a higher level of multidrug resistance, which is likely connected with a higher level of conjugation events (53). Our analysis also indicated a mild negative association between functional T6SS^i2^ and MDR across *E. coli* genomes (Fig. 6). These two factors combined (i.e., increased stress resistance and MDR acquisition through enhanced horizontal gene transfer) could contribute to the global pathogenic success of ST131.

Our co-occurrence analysis revealed that T6SS^i1^ mostly clustered with IPEC VAGs, while T6SS^i2^ largely clustered with ExPEC VAGs (Fig. 6). T6SS^i1^ occurrence correlated positively with many typical IPEC VAGs such as *stx1* and *stx2*, or T3SS-related genes such as *escC* (2, 5), and T6SS^i2^ occurrence correlated positively with typical ExPEC markers such as capsule or yersiniabactin (3). In general, we did not observe clustering of IPEC together with ExPEC VAGs. Their co-occurrence analysis also displayed negative correlations, indicating that both these pathogroups are, in the majority of cases, mutually exclusive. Furthermore, our PCA of T6SS-positive genomes confirmed this VAGs-based differentiation between ExPEC and IPEC strains (Fig. 7A and B). Within each of these groups, we also observed smaller subclusters, hinting at the existence of genome subgroups with distinct properties (that likely reflect the different environmental contexts from which these strains were isolated). Importantly, this type of global pattern could only be identified through our merged database of *E. coli* T6SS^i^ regions that combines input from multiple sources, given that different sources showed different distribution patterns for T6SS subclasses.

While genomic information related to the presence of T6SSs and VAGs could be successfully employed to distinguish between IPEC- and ExPEC-associated genomes, including a separate category for commensals proved more difficult. Several reasons likely underlie this phenomenon. First, we only had a limited number of genomes in our entire data set that could be categorized as commensals (571 genomes labeled as Healthy-Community and 183 as Non-Pathogen). Second, source metadata are somewhat unreliable for determining true commensals, as healthy humans and animals can carry ExPEC strains in their guts without any apparent symptoms. Similarly, ExPEC strains can be isolated from the environment, rendering a source-based assignment of commensals vs pathogens also unreliable. Third, the question remains whether true consistent commensals really exist. Our VAGs-based PCA analysis revealed that even genomes that could be categorized as commensals clustered among ExPEC (Fig. 7A and B), suggesting their pathogenic manifestation could be context- and/or host-dependent.

In conclusion, our large-scale analysis underscores the remarkable genomic diversity of *E. coli* and reveals distinct patterns in the distribution of T6SS^i^ subclasses across different phylogenetic groups and clinical contexts. By integrating extensive genomic insights, we demonstrate that IPEC and ExPEC-associated genomes can be confidently distinguished based on their genetic profiles, providing a robust framework for predicting the pathogenic potential of unknown strains. Furthermore, our findings challenge certain long-standing assumptions, emphasizing the necessity of large-scale studies before extrapolating smaller-scale observations to the broader *E. coli* population. Beyond these insights, our annotated *E. coli* genome collection stands as a valuable resource for future research, enabling deeper exploration of clinically relevant genetic elements.

## MATERIALS AND METHODS

### Genomic data collection and annotation

We downloaded a total of 136,051 available genomes with metadata from EB from February to April 2023 (27, 74). To avoid duplications, each genome represented a unique Uberstrain (27). Each Uberstrain can be a parent to one or more essentially identical substrains, and these substrains were not included in our collection. In addition, we downloaded three types of metadata data sets from EB: Assembly stats, Phylotype, and Annotation, and merged them using Uberstrain as a barcode to match these data sets. We then used an Assembly barcode (and relabeled it as Barcode) to match the metadata with the results of the evaluation of the genomic sequences. The metadata data set Assembly stats specifies basic genomic quality metrics, such as such as Coverage, N50, Length, Contig Number, and Low-Quality Bases, and we kept them as part of Table S1. To determine phylogenetic groups, we used the ClermonTyping tool (https://github.com/A-BN/ClermonTyping), and we merged our results with EB metadata (EB also provides a phylogenetic group assignment based on either ClermonTyping [54] or EZClermont [75] in the Phylotype metadata sheet [27]). We filtered out genomes that showed irregular PG or species results (269 genomes) or were detected as part of very minor clades III, IV, or V (total of 65 genomes). We filtered out additional genomes (4,107) due to low coverage (below 30×), resulting in a final data set of 131,610 genomes.

Using the existing EB metadata, we provided additional annotations (Table S1) by creating four columns, Source curated, ExPEC type, IPEC type*,* and Gender, and updating information in the columns Source Pathogen (in EB originally called Path/Nonpath) and Source Pathogen details (originally Simple Patho) as these occasionally contain information not relevant for our purposes (e.g., AMR profile).

Source curated divided the genomes into eight categories: Clinical (1,136 genomes), ExPEC (20,523 genomes), Healthy-Community (571 genomes), IPEC (42,791 genomes), Mixed (159 genomes), ND (not determined) (65,828 genomes), Non Pathogen (183 genomes), and Pathogen (419 genomes). The category Clinical contains genomes with keywords Hospital, Clinical, or Patient in their metadata, which thus likely correspond to pathogenic strains, but where we lack the necessary information to reliably divide them into the IPEC or ExPEC category. The ExPEC category was assigned based on existing metadata, if ExPEC was already specified in the Source Pathogen or Source Pathogen details, Simple Disease*,* or Disease information, or if the source of isolation was ExPEC-relevant (e.g., urine, blood). For other sources (e.g., skin, eyes, ears), infection needed to be mentioned to assign the ExPEC label. The Healthy-Community category contains genomes for which it was specifically mentioned that they originated from healthy individuals (humans and animals) or from the community (“healthy” or “community”). The assignment of genomes to the IPEC category was mainly based on Pathovar predictions using the established virulence scheme (recovered from the EB data set Phylotype). A smaller part of the IPEC assignment was based on the direct contributor’s metadata. The Mixed category contains genomes for which both the ExPEC and IPEC label would be applicable (e.g., a genome with predicted IPEC pathovar isolated from a urinary infection). Genomes for which further annotation was not possible based on the provided metadata were classified as ND. The Non Pathogen category contains genomes for which this information was specified by the EB contributors (“non-pathogen”) and no other apparently contradicting metadata exists. Similarly, the Pathogen category contains genomes for which this information was provided by the contributors (“pathogen”) and no other metadata could link these genomes to the IPEC or ExPEC pathogroup. For the Clinical, ND, and Pathogen category, we expect pathogenic strains to be enriched in ExPEC genomes, given that IPEC can be identified based on their genomic information and a well-defined VAGs scheme.

The columns ExPEC type and IPEC type (Table S1) further divide these two pathogroups into specific pathovars or other defined groups. In the case of ExPEC, we deviated from the classically defined pathovars due to unreliability of the assignment and an underrepresentation in some cases (e.g., neonatal meningitis-causing *E. coli* [NMEC] was specified for only four strains in the collection, and only 26 additional strains were linked to the brain). Our division of ExPEC type contained five categories: UPEC (12,346 genomes, linked to urine or UTIs), BSI (4,957 genomes, linked to blood), Respiratory (407 genomes, linked to respiratory infection or intubation), APEC (446 genomes, linked to avian ExPEC), and other unspecified ExPEC (2,524 genomes, other sources). If there was not sufficient metadata to reliably determine whether a genome was ExPEC-linked or not, genomes were assigned to the category NS (68,322 genomes). If a genome was already linked to an IPEC pathotype, and thus likely not ExPEC, it was assigned to the category No (42,608 genomes). IPEC type was, in the majority of cases (>97%), assigned based on the virulence scheme of genomes (Table S1). The pathovars included EHEC (24,298 genomes), STEC (9,966 genomes), EPEC (5,176 genomes), ETEC (2,266 genomes), EIEC (356 genomes) and hybrid groups of ETEC/STEC (557 genomes), ETEC/EHEC (102 genomes), and ETEC/EPEC (46 genomes). All remaining strains for which the virulence scheme showed negative results were assigned into the No category (88,342 genomes), with the exception of strains for which the virulence scheme results were missing, which are grouped in the ND category (501 genomes). The column Gender is specified in the rare instances where the genome originated from a female- or male-specific source (e.g., vagina, uterus, semen) or when it was specifically mentioned by the EB contributors.

Further metadata adjustments included adding missing Continent information based on Country and adding Source Niche data where possible from Source type or Source details. There were only two genomes with the original Continent label Central America (Uberstrains ESC_GA0177AA and ESC_GA0176AA), which were merged with North America. For Source Niche, Aquatic Animal (603 genomes) was merged with Aquatic (one genome, Uberstrain ESC_RA0094AA, which also came from an aquatic animal). The merged group was named Aquatic.

### Screen for T6SS components

To construct a comprehensive sequence database of *E. coli* T6SS components, we used multiple sources as genomic references of T6SS components of different T6SS classes and subclasses. We always used ABRicate (https://github.com/tseemann/abricate) to screen for the presence of T6SS-associated genes with a threshold of 85% for both query coverage and identity. We chose an 85% threshold to remain consistent with (53), which represented the largest-scale evaluation of T6SS in *E. coli* before our study. A first source came from a screen for ExPEC-associated regions, where we identified a T6SS in a strain of ST131 (KO_178_B, originally isolated in reference 76), BioSample SAMN35728132) (76). This same region was identified in a previous study (53) and showed only one SNP difference with ours. To investigate the broader occurrence of various T6SS^i^ subclasses, we downloaded sequences of 10 T6SS regions identified by Ma et al. (47), who identified the *E. coli*-related subclasses T6SS^i1^, T6SS^i2^, and T6SS^i4b^ across APEC genomes. When necessary, we provided additional annotations to assign genes as *tssA–tssM* using BLAST (77) or the SecReT6 database (https://bioinfo-mml.sjtu.edu.cn/SecReT6/index.php) (78) (Table S2). Given that this approach did not always find all 13 core components within identified T6SS genomic regions, we also implemented all reported functional *E. coli* T6SS regions from the SecReT6 database. We evaluated both the completeness and presence of the three T6SS subclasses. We defined Present/Presence (P) as detecting at least 12 core genes from a respective subclass, while Complete/Completeness (C) corresponds to the detection of all 13 core genes. We performed this analysis using our merged database, but also separately using only the sequences from Ma & KO_178_B (DB1) or SecReT6 (DB2) (Fig. S3 to S7).

To explore differences in the prevalence of experimentally validated T6SS^i^-associated regulatory genes in *E. coli*, we created a database by extracting these genes from SecReT6. We then used ABRicate to screen for their presence with a threshold of 85% for both query coverage and identity thresholds.

### Phylogeny of the most prevalent ST representatives

Across our genome collection, we identified 35 prevalent STs (using a cutoff of ≥500 genomes for a given ST). We pseudo-randomly selected one genome per ST (i.e., typically one of the first genomes per ST in alphabetical order by Assembly barcode with the intention to include diverse countries and sources of origin) and used it to build a phylogenetic tree to visualize the prevalence of T6SSs in the approximate phylogenetic context. The genomes used to build the tree are specified in Fig. S1. The phylogenetic analysis was based on Prokka (v1.14.5) open reading frames prediction (79), and Roary (v3.7.0) (80) multi-fasta alignment. RAxML (81), supported by 100 bootstraps, was used to calculate the tree. The prevalence of individual T6SS^i^ subclasses was examined in terms of both their Presence and Completeness. In addition, we separately inspected STs with large differences between both categories by checking the prevalence of individual genes and identifying those that were missing (and thus responsible for the discrepancy between Presence and Completeness). For each such ST, we examined an individual genome sequence and visually inspected the T6SS region to uncover the potential reason underlying the disappearance of the individual gene.

### Virulence Factor Database (VFDB) and ResFinder screen

We used the VFDB (http://www.mgc.ac.cn/VFs/) (61) to screen for the presence of known VAGs in our *E. coli* genome collection using ABRicate with a 90% threshold for both query coverage and identity. We chose this threshold as it is commonly used with the VFDB (82–84). We compared the obtained list with the distribution pattern of T6SS and examined potential correlations in their mutual presence or absence. To obtain meaningful results, we limited this screen to VAGs that are found in more than 3% and less than 90% of genomes in our collection. This resulted in a list of 242 VAGs (from a total of 404 that were originally identified). The prevalence of these 242 VAGs for the 35 dominant STs is summarized in Fig. S1. For our correlation analysis, we filtered out T6SS-relevant genes within the VFDB, which were found to mostly associate with T6SS^i1^, to avoid artificial positive correlations. This resulted in a total of 227 VAGs that were considered for the global correlation analysis. To examine the potential co-occurrence of T6SS with MDR, we used ResFinder (v.3.0) (85) to screen for the presence of ARGs. We filtered out *mdf*(*A*), given that it is normally present in *E. coli* (86, 87), and then we calculated the sum of overall detected ARGs. We defined the MDR on multiple levels, starting from genomes with minimally three ARGs and ending with genomes with minimally ten ARGs, yielding eight MDR groups in total, marked MDR3-MDR10. The correlations of presence/absence of T6SS, VAGs, and MDR were calculated using polychoric correlation coefficients. The code for this is available at https://github.com/Govers-Lab/Nesporova_and_Govers_2024.

### Prediction of pathogenic groups based on T6SSs and virulence-associated genes

Multiple machine learning models (random forest [88], logistic regression [89], K-nearest neighbors [90], naive Bayes [91], gradient boosting [92], extra trees [93], decision tree [94], and a simple neural network [95]) were trained with an 80-20 train-test split, and a 5-fold cross-validation to tune the hyperparameters for each model. The performance metrics of the best-performing models after hyperparameter tuning can be found in Table S12. Although most models achieved high classification accuracy (test accuracy > 0.98), the extra trees classifier was ultimately selected to predict the pathogroups of the ND class. This choice was based on its strong predictive performance and its capability to assess feature importance, enabling further insight into the role of specific genes in distinguishing between ExPEC and IPEC. The prediction results are stored in Table S13 in column Source.predicted, where the ND class will either have a label ND_IPEC or ND_ExPEC assigned depending on the output of the model.

### Statistical analysis and data visualization

We used RStudio (v2023.06.0) with R (v4.4.1) for data merge, summary, statistical analysis, and figure creation, except for the principal component analysis (PCA) (pca_T6SS.ipynb) and machine learning models (pathogenic_group_prediction.ipynb) which were written in Python (v3.10.12) using libraries including pandas, NumPy, Matplotlib, and scikit-learn. All code is specified and explained at https://github.com/Govers-Lab/Nesporova_and_Govers_2024 together with raw and intermediate data. We used the pairwise Wilcoxon test to calculate the significance (α = 0.05) of the pairwise differences between specific groups with Benjamini-Hochberg adjustment of the *P*-values. We used Geneious (v7.1.9) to check annotations and alignment of reference sequences and specific genomic regions. The phylogenetic tree was visualized using the Interactive Tree of Life (iTOL v6) (96).

## Data Availability

All data generated or analyzed during this study are included in this published article and its supplemental files.

## SUPPLEMENTAL MATERIAL

Supplemental Material msystems.00105-25-s0001.pdf Supplemental figures and table legends.

Table S1, part 1 msystems.00105-25-s0002.xlsb Original EnteroBase metadata data sets, metadata added or curated in this study, and results of our screen for T6SSi and VFDB.

Table S1, part 2 msystems.00105-25-s0003.xlsb Original EnteroBase metadata data sets, metadata added or curated in this study, and results of our screen for T6SSi and VFDB.

Table S1, part 3 msystems.00105-25-s0004.xlsb Original EnteroBase metadata data sets, metadata added or curated in this study, and results of our screen for T6SSi and VFDB.

Supplemental Tables msystems.00105-25-s0005.xlsx Table S2 to S14.

ASM does not own the copyrights to Supplemental Material that may be linked to, or accessed through, an article. The authors have granted ASM a non-exclusive, world-wide license to publish the Supplemental Material files. Please contact the corresponding author directly for reuse.
