# Supplementary material for: Large-scale genomic analysis reveals the distribution and diversity of type VI secretion systems in Escherichia coli
Source: mSystems. 2025 Jun 18;10(7):e00105-25. doi: 10.1128/msystems.00105-25 (PMC12282107; doi:10.1128/msystems.00105-25)
Supplement: Supplemental Material — Supplemental figures and table legends. [file msystems.00105-25-s0001.pdf]

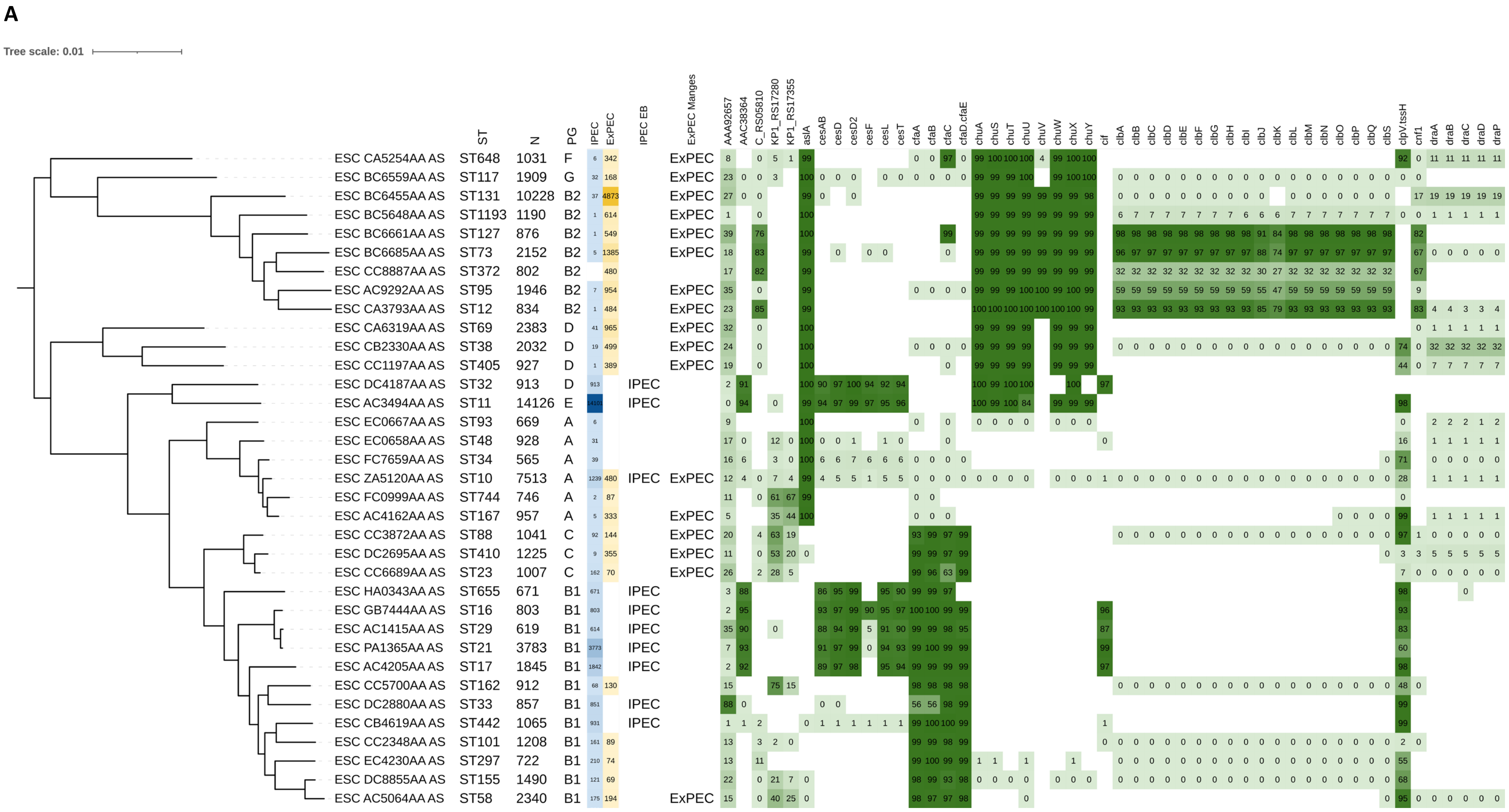
A
Tree scale: 0.01
ST
N
PG
IPEC
ExPEC
IPEC EB
ExPEC Manges
AAA92657
AAC38364
C_RS05810
KP1_RS17280
KP1_RS17355
aslA
cesAB
cesD
cesD2
cesF
cesL
cesT
cfaA
cfaB
cfaC
cfaD.cfaE
chuA
chuS
chuT
chuU
chuV
chuW
chuX
chuY
cif
clbA
clbB
clbC
clbD
clbE
clbF
clbG
clbH
clbI
clbJ
clbK
clbL
clbM
clbN
clbO
clbP
clbQ
clbS
clpV.tssH
cnf1
draA
draB
draC
draD
draP
ESC CA5254AA AS ST648 1031 F
ESC BC6559AA AS ST117 1909 G
ESC BC6455AA AS ST131 10228 B2
ESC BC5648AA AS ST1193 1190 B2
ESC BC6661AA AS ST127 876 B2
ESC BC6685AA AS ST73 2152 B2
ESC CC8887AA AS ST372 802 B2
ESC AC9292AA AS ST95 1946 B2
ESC CA3793AA AS ST12 834 B2
ESC CA6319AA AS ST69 2383 D
ESC CB2330AA AS ST38 2032 D
ESC CC1197AA AS ST405 927 D
ESC DC4187AA AS ST32 913 D
ESC AC3494AA AS ST11 14126 E
ESC EC0667AA AS ST93 669 A
ESC EC0658AA AS ST48 928 A
ESC FC7659AA AS ST34 565 A
ESC ZA5120AA AS ST10 7513 A
ESC FC0999AA AS ST744 746 A
ESC AC4162AA AS ST167 957 A
ESC CC3872AA AS ST88 1041 C
ESC DC2695AA AS ST410 1225 C
ESC CC6689AA AS ST23 1007 C
ESC HA0343AA AS ST655 671 B1
ESC GB7444AA AS ST16 803 B1
ESC AC1415AA AS ST29 619 B1
ESC PA1365AA AS ST21 3783 B1
ESC AC4205AA AS ST17 1845 B1
ESC CC5700AA AS ST162 912 B1
ESC DC2880AA AS ST33 857 B1
ESC CB4619AA AS ST442 1065 B1
ESC CC2348AA AS ST101 1208 B1
ESC EC4230AA AS ST297 722 B1
ESC DC8855AA AS ST155 1490 B1
ESC AC5064AA AS ST58 2340 B1

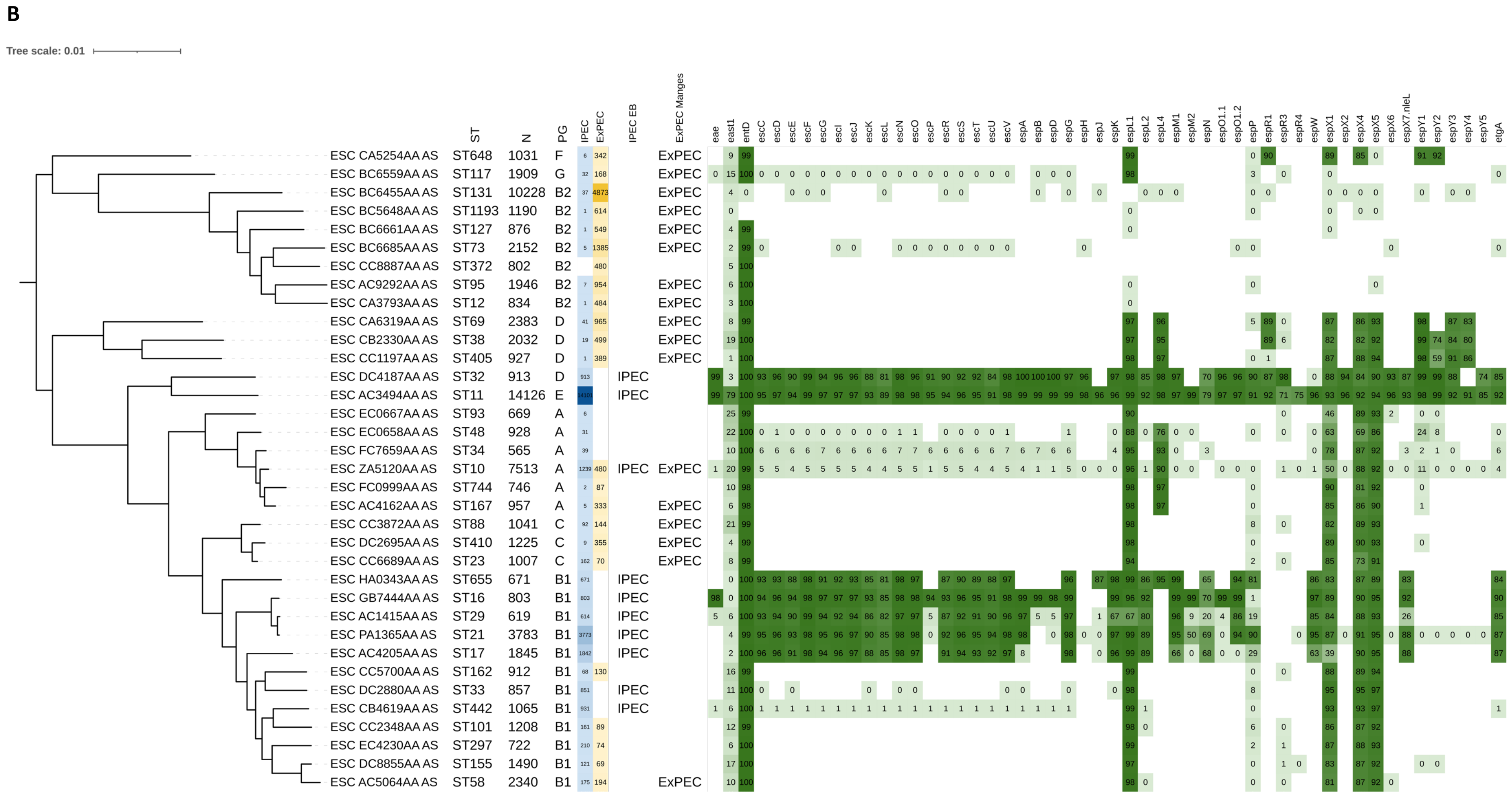
B
Tree scale: 0.01
ST
N
PG
IPEC
ExPEC
IPEC EB
ExPEC Manges
eae east1 entD escC escD escE escF escG escI escJ escK escL escN escO escP escR escS escT escU escV espA espB espD espG espH espJ espK espL1 espL2 espL4 espM1 espM2 espN espO1.1 espO1.2 espP espR1 espR3 espR4 espW espX1 espX2 espX4 espX5 espX6 espX7.nleL espY1 espY2 espY3 espY4 espY5 etgA
ESC CA5254AA AS ST648 1031 F ExPEC
ESC BC6559AA AS ST117 1909 G ExPEC
ESC BC6455AA AS ST131 10228 B2 ExPEC
ESC BC5648AA AS ST1193 1190 B2 ExPEC
ESC BC6661AA AS ST127 876 B2 ExPEC
ESC BC6685AA AS ST73 2152 B2 ExPEC
ESC CC8887AA AS ST372 802 B2
ESC AC9292AA AS ST95 1946 B2 ExPEC
ESC CA3793AA AS ST12 834 B2 ExPEC
ESC CA6319AA AS ST69 2383 D ExPEC
ESC CB2330AA AS ST38 2032 D ExPEC
ESC CC1197AA AS ST405 927 D ExPEC
ESC DC4187AA AS ST32 913 D IPEC
ESC AC3494AA AS ST11 14126 E IPEC
ESC EC0667AA AS ST93 669 A
ESC EC0658AA AS ST48 928 A
ESC FC7659AA AS ST34 565 A
ESC ZA5120AA AS ST10 7513 A IPEC ExPEC
ESC FC0999AA AS ST744 746 A
ESC AC4162AA AS ST167 957 A ExPEC
ESC CC3872AA AS ST88 1041 C ExPEC
ESC DC2695AA AS ST410 1225 C ExPEC
ESC CC6689AA AS ST23 1007 C ExPEC
ESC HA0343AA AS ST655 671 B1 IPEC
ESC GB7444AA AS ST16 803 B1 IPEC
ESC AC1415AA AS ST29 619 B1 IPEC
ESC PA1365AA AS ST21 3783 B1 IPEC
ESC AC4205AA AS ST17 1845 B1 IPEC
ESC CC5700AA AS ST162 912 B1
ESC DC2880AA AS ST33 857 B1 IPEC
ESC CB4619AA AS ST442 1065 B1 IPEC
ESC CC2348AA AS ST101 1208 B1
ESC EC4230AA AS ST297 722 B1
ESC DC8855AA AS ST155 1490 B1
ESC AC5064AA AS ST58 2340 B1 ExPEC

C

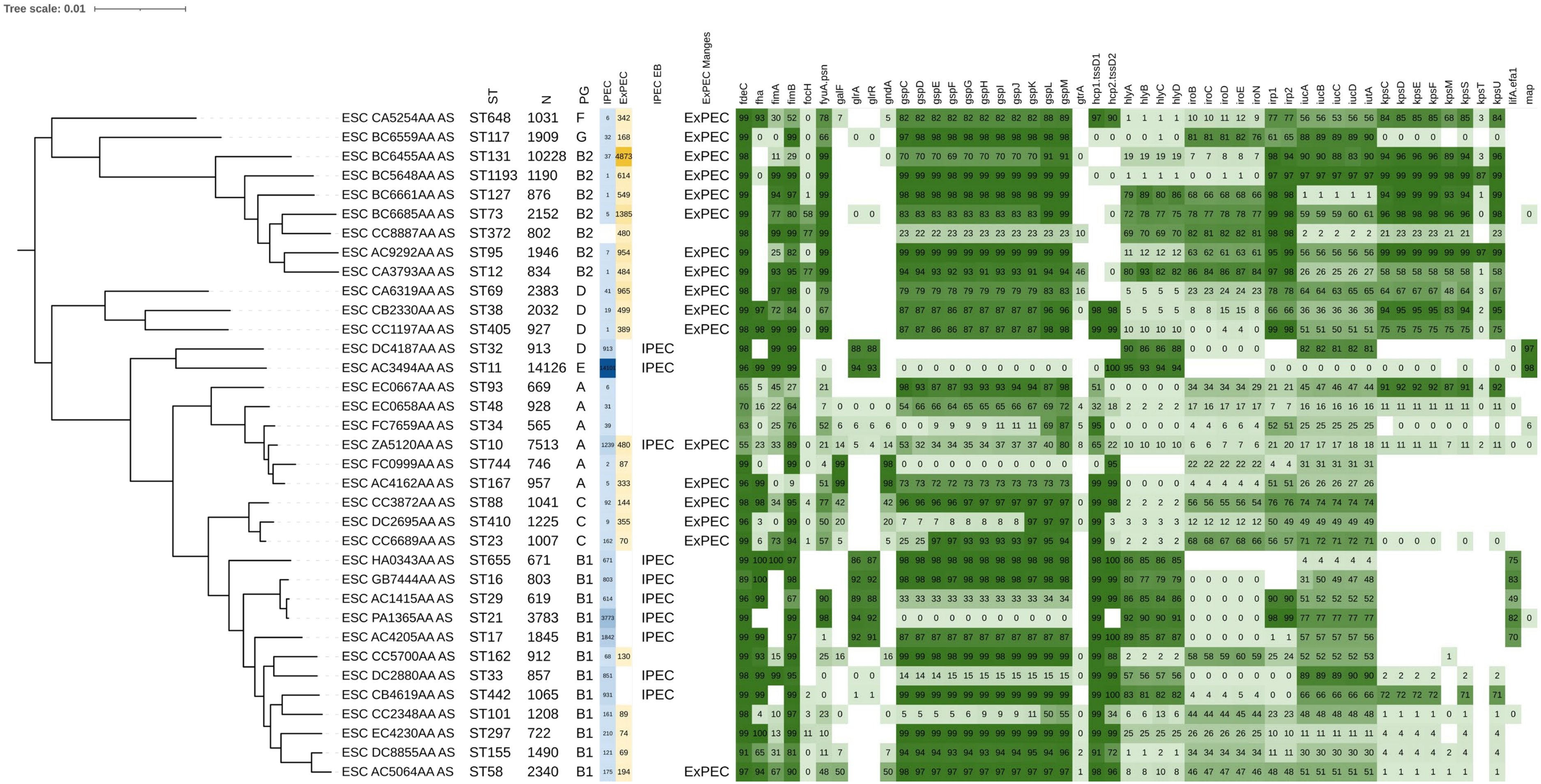

Tree scale: 0.01
ST
N
PG
IPEC
ExPEC
IPEC EB
ExPEC Manges
fdeC fha fimA fimB focH fyuA.psn galF glrA glrR gndA gspC gspD gspE gspF gspG gspH gspI gspJ gspK gspL gspM gtrA hcp1.tssD1 hcp2.tssD2 hlyA hlyB hlyC hlyD iroB iroC iroD iroE iroN irp1 irp2 iucA iucB iucC iucD iutA kpsC kpsD kpsE kpsF kpsM kpsS kpsT kpsU lifA.efa1 map
ESC CA5254AA AS ST648 1031 F ExPEC
ESC BC6559AA AS ST117 1909 G ExPEC
ESC BC6455AA AS ST131 10228 B2 ExPEC
ESC BC5648AA AS ST1193 1190 B2 ExPEC
ESC BC6661AA AS ST127 876 B2 ExPEC
ESC BC6685AA AS ST73 2152 B2 ExPEC
ESC CC8887AA AS ST372 802 B2
ESC AC9292AA AS ST95 1946 B2 ExPEC
ESC CA3793AA AS ST12 834 B2 ExPEC
ESC CA6319AA AS ST69 2383 D ExPEC
ESC CB2330AA AS ST38 2032 D ExPEC
ESC CC1197AA AS ST405 927 D ExPEC
ESC DC4187AA AS ST32 913 D IPEC
ESC AC3494AA AS ST11 14126 E IPEC
ESC EC0667AA AS ST93 669 A
ESC EC0658AA AS ST48 928 A
ESC FC7659AA AS ST34 565 A
ESC ZA5120AA AS ST10 7513 A IPEC ExPEC
ESC FC0999AA AS ST744 746 A
ESC AC4162AA AS ST167 957 A ExPEC
ESC CC3872AA AS ST88 1041 C ExPEC
ESC DC2695AA AS ST410 1225 C ExPEC
ESC CC6689AA AS ST23 1007 C ExPEC
ESC HA0343AA AS ST655 671 B1 IPEC
ESC GB7444AA AS ST16 803 B1 IPEC
ESC AC1415AA AS ST29 619 B1 IPEC
ESC PA1365AA AS ST21 3783 B1 IPEC
ESC AC4205AA AS ST17 1845 B1 IPEC
ESC CC5700AA AS ST162 912 B1
ESC DC2880AA AS ST33 857 B1 IPEC
ESC CB4619AA AS ST442 1065 B1 IPEC
ESC CC2348AA AS ST101 1208 B1
ESC EC4230AA AS ST297 722 B1
ESC DC8855AA AS ST155 1490 B1
ESC AC5064AA AS ST58 2340 B1 ExPEC

D

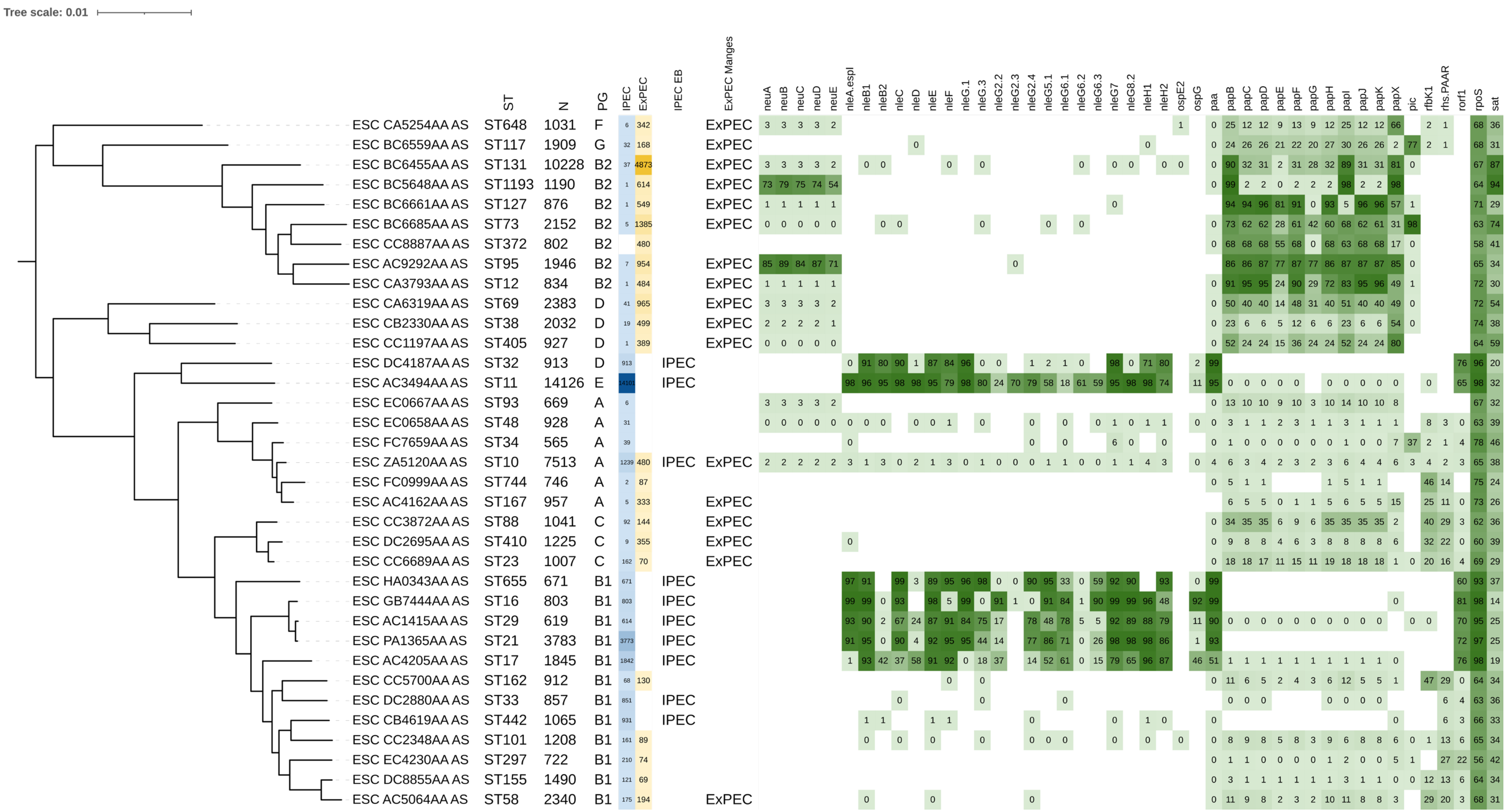
Tree scale: 0.01
ST
N
PG
IPEC
ExPEC
IPEC EB
ExPEC Manges
neuA neuB neuC neuD neuE nleA.espI nleB1 nleB2 nleC nleD nleE nleF nleG.1 nleG.3 nleG2.2 nleG2.3 nleG2.4 nleG5.1 nleG6.1 nleG6.2 nleG6.3 nleG7 nleG8.2 nleH1 nleH2 ospE2 ospG paa papB papC papD papE papF papG papH papI papJ papK papX pic rfbK1 rhs.PAAR rorf1 rpoS sat
ESC CA5254AA AS ST648 1031 F ExPEC
ESC BC6559AA AS ST117 1909 G ExPEC
ESC BC6455AA AS ST131 10228 B2 ExPEC
ESC BC5648AA AS ST1193 1190 B2 ExPEC
ESC BC6661AA AS ST127 876 B2 ExPEC
ESC BC6685AA AS ST73 2152 B2 ExPEC
ESC CC8887AA AS ST372 802 B2
ESC AC9292AA AS ST95 1946 B2 ExPEC
ESC CA3793AA AS ST12 834 B2 ExPEC
ESC CA6319AA AS ST69 2383 D ExPEC
ESC CB2330AA AS ST38 2032 D ExPEC
ESC CC1197AA AS ST405 927 D ExPEC
ESC DC4187AA AS ST32 913 D IPEC
ESC AC3494AA AS ST11 14126 E IPEC
ESC EC0667AA AS ST93 669 A
ESC EC0658AA AS ST48 928 A
ESC FC7659AA AS ST34 565 A
ESC ZA5120AA AS ST10 7513 A IPEC ExPEC
ESC FC0999AA AS ST744 746 A
ESC AC4162AA AS ST167 957 A ExPEC
ESC CC3872AA AS ST88 1041 C ExPEC
ESC DC2695AA AS ST410 1225 C ExPEC
ESC CC6689AA AS ST23 1007 C ExPEC
ESC HA0343AA AS ST655 671 B1 IPEC
ESC GB7444AA AS ST16 803 B1 IPEC
ESC AC1415AA AS ST29 619 B1 IPEC
ESC PA1365AA AS ST21 3783 B1 IPEC
ESC AC4205AA AS ST17 1845 B1 IPEC
ESC CC5700AA AS ST162 912 B1
ESC DC2880AA AS ST33 857 B1 IPEC
ESC CB4619AA AS ST442 1065 B1 IPEC
ESC CC2348AA AS ST101 1208 B1
ESC EC4230AA AS ST297 722 B1
ESC DC8855AA AS ST155 1490 B1
ESC AC5064AA AS ST58 2340 B1 ExPEC

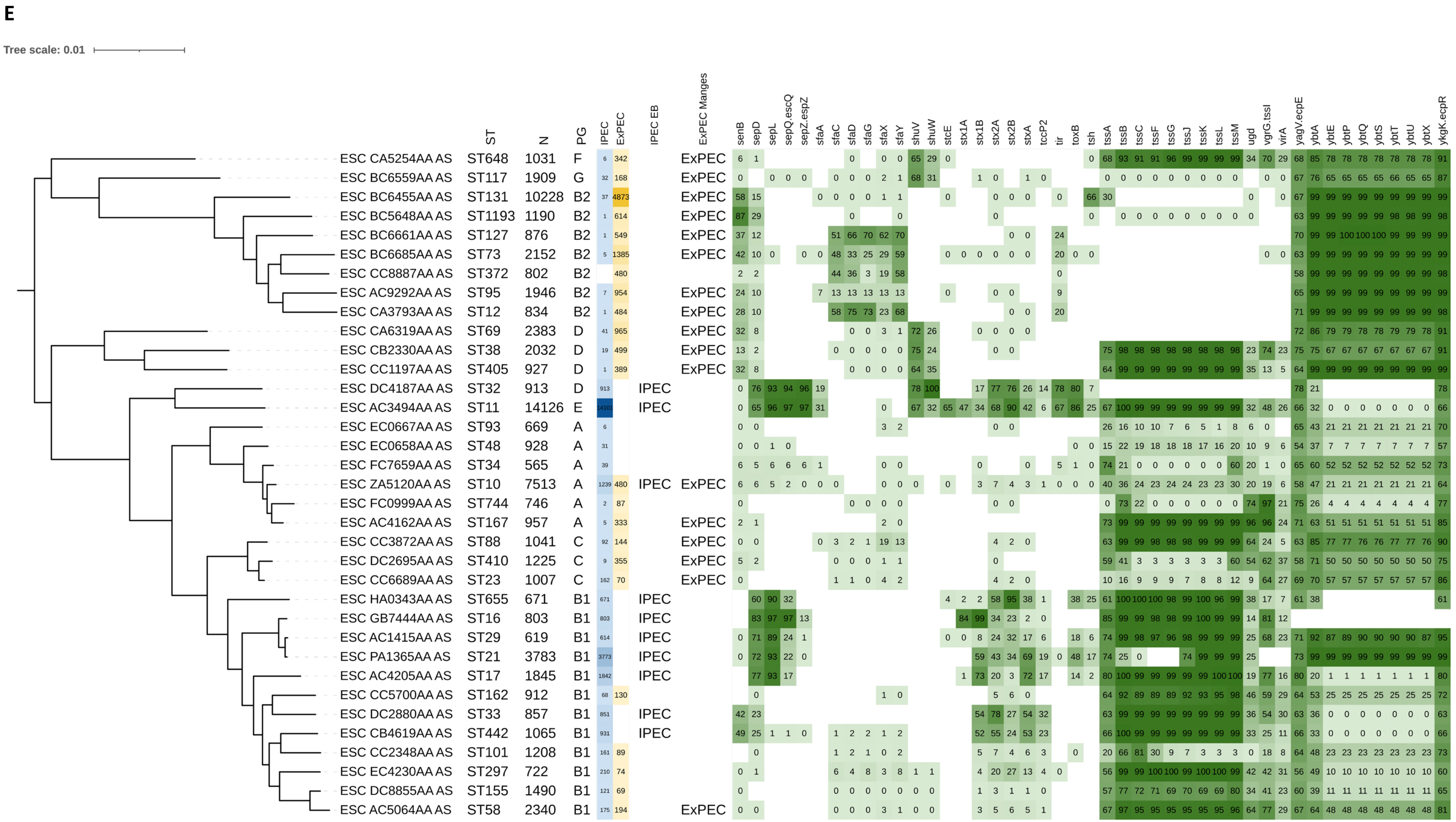
E
Tree scale: 0.01
ST
N
PG
IPEC
ExPEC
IPEC EB
ExPEC Manges
senB sepD sepL sepQ.escQ sepZ.espZ sfaA sfaC sfaD sfaG sfaX sfaY shuV shuW stcE stx1A stx1B stx2A stx2B stxA tccP2 tir toxB tsh tssA tssB tssC tssF tssG tssJ tssK tssL tssM ugd vgrG.tssI virA yagV.ecpE ybtA ybtE ybtP ybtQ ybtS ybtT ybtU ybtX ykgK.ecpR
ESC CA5254AA AS ST648 1031 F ExPEC
ESC BC6559AA AS ST117 1909 G ExPEC
ESC BC6455AA AS ST131 10228 B2 ExPEC
ESC BC5648AA AS ST1193 1190 B2 ExPEC
ESC BC6661AA AS ST127 876 B2 ExPEC
ESC BC6685AA AS ST73 2152 B2 ExPEC
ESC CC8887AA AS ST372 802 B2
ESC AC9292AA AS ST95 1946 B2 ExPEC
ESC CA3793AA AS ST12 834 B2 ExPEC
ESC CA6319AA AS ST69 2383 D ExPEC
ESC CB2330AA AS ST38 2032 D ExPEC
ESC CC1197AA AS ST405 927 D ExPEC
ESC DC4187AA AS ST32 913 D IPEC
ESC AC3494AA AS ST11 14126 E IPEC
ESC EC0667AA AS ST93 669 A
ESC EC0658AA AS ST48 928 A
ESC FC7659AA AS ST34 565 A
ESC ZA5120AA AS ST10 7513 A IPEC ExPEC
ESC FC0999AA AS ST744 746 A
ESC AC4162AA AS ST167 957 A ExPEC
ESC CC3872AA AS ST88 1041 C ExPEC
ESC DC2695AA AS ST410 1225 C ExPEC
ESC CC6689AA AS ST23 1007 C ExPEC
ESC HA0343AA AS ST655 671 B1 IPEC
ESC GB7444AA AS ST16 803 B1 IPEC
ESC AC1415AA AS ST29 619 B1 IPEC
ESC PA1365AA AS ST21 3783 B1 IPEC
ESC AC4205AA AS ST17 1845 B1 IPEC
ESC CC5700AA AS ST162 912 B1
ESC DC2880AA AS ST33 857 B1 IPEC
ESC CB4619AA AS ST442 1065 B1 IPEC
ESC CC2348AA AS ST101 1208 B1
ESC EC4230AA AS ST297 722 B1
ESC DC8855AA AS ST155 1490 B1
ESC AC5064AA AS ST58 2340 B1 ExPEC

Supplementary Figure S1. Phylogenetic analysis of 35 most dominant STs. The labels of the genomes correspond to the Assembly Barcode in EnteroBase and the Barcode in our data (Supplementary Table 1). The metadata included in our phylogenetic tree are sequence types (**ST**), the number of genomes belonging to the respective ST in our collection (**N**), phylogenetic group (**PG**), heatmap (in blue) highlighting the dominant IPEC STs (threshold of min. 50 IPEC genomes) (**IPEC**), heatmap (in yellow) highlighting the dominant ExPEC STs in our study (threshold of min. 50 ExPEC genomes), IPEC label given to STs which belonged to the 20 dominant IPEC STs determined based on EnteroBase information (**IPEC EnteroBase**), ExPEC label given to 20 dominant ExPEC STs undertaken from[15] (**ExPEC Manges**), and a heatmap (in green) highlighting the prevalence of 242 VAGs in the dominant STs. The heatmap is divided into 5 separate figures (**A-E**) to ensure readability.

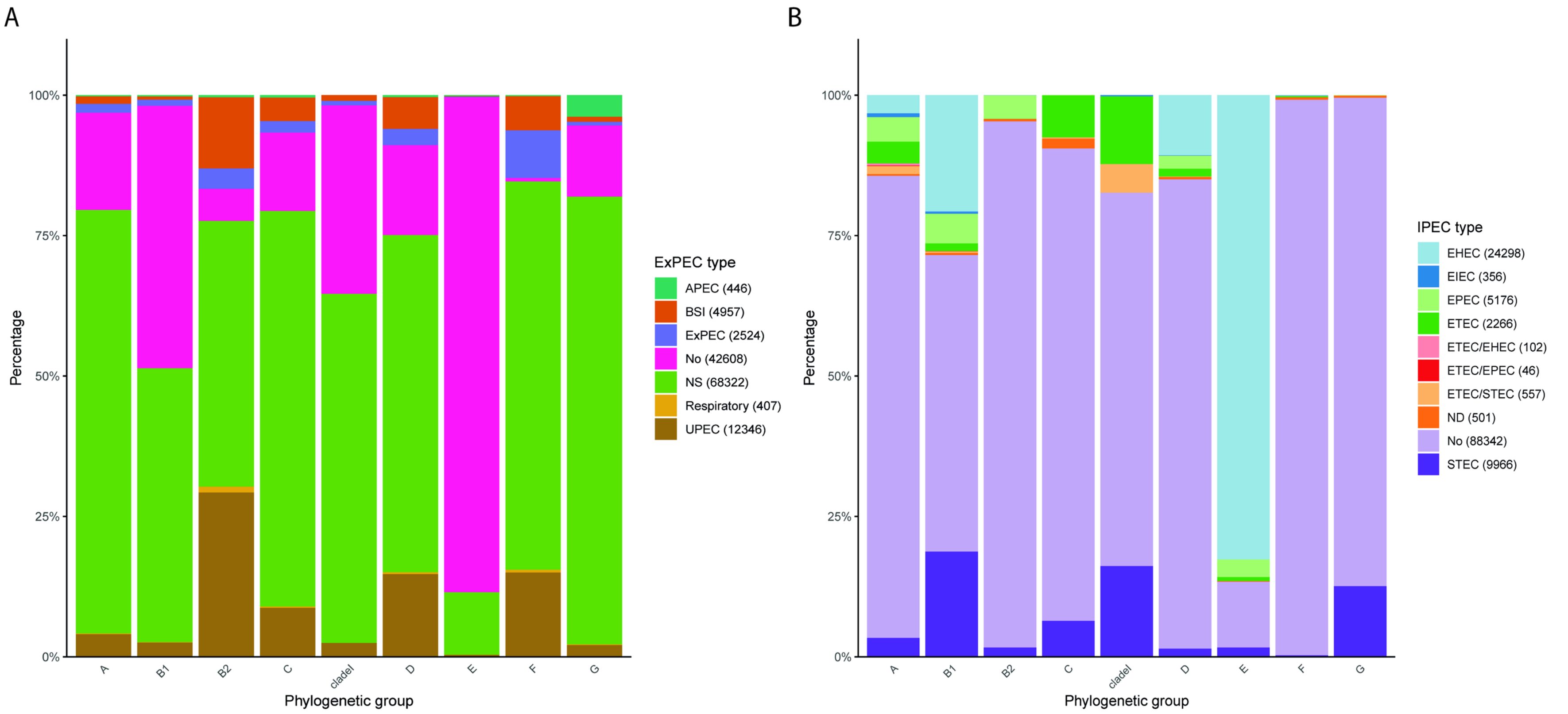

Supplementary Figure S2. Distribution of ExPEC type/IPEC types across phylogenetic groups. A. Bar graphs showing the relative distribution of ExPEC types across major phylogenetic groups. **B.** Bar graphs showing the relative distribution of IPEC types across major phylogenetic groups.

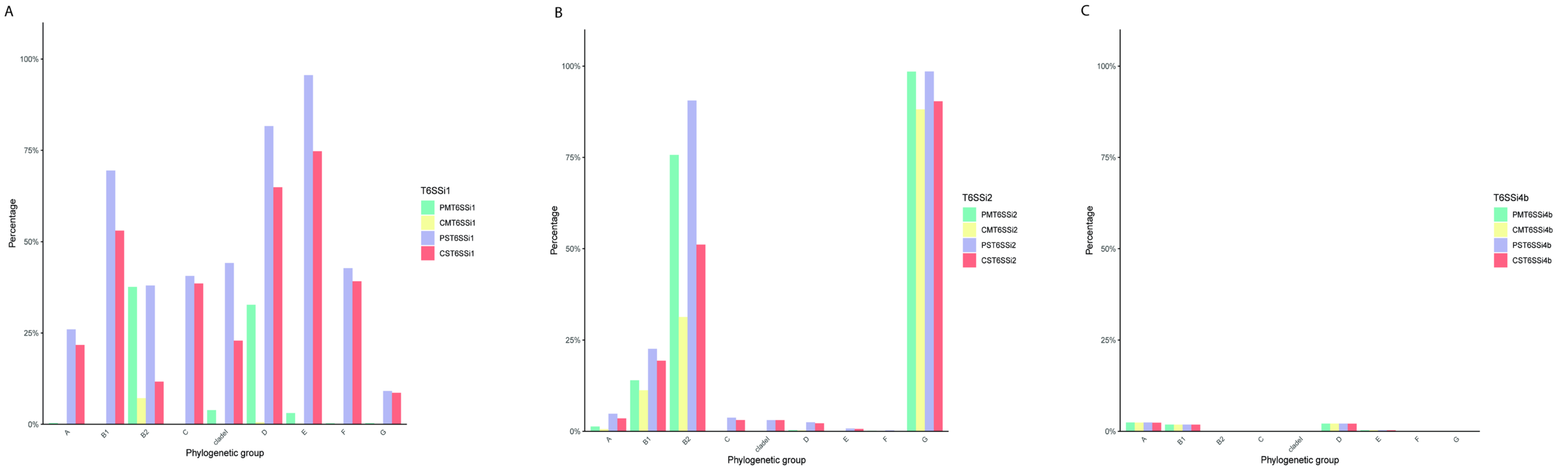

Supplementary Figure S3. Relative prevalence of each $T6SS^{i}$ subclass across phylogenetic groups for both databases used a source for T6SS components. The panels show the prevalence of $T6SS^{i1}$ (**A**), $T6SS^{i2}$ (**B**) and $T6SS^{i4}$ (**C**) across phylogenetic groups considering *Presence* (marked with P) and *Completeness* (marked with C) for both databases, DB1 (marked with M) and DB2 (marked with S).

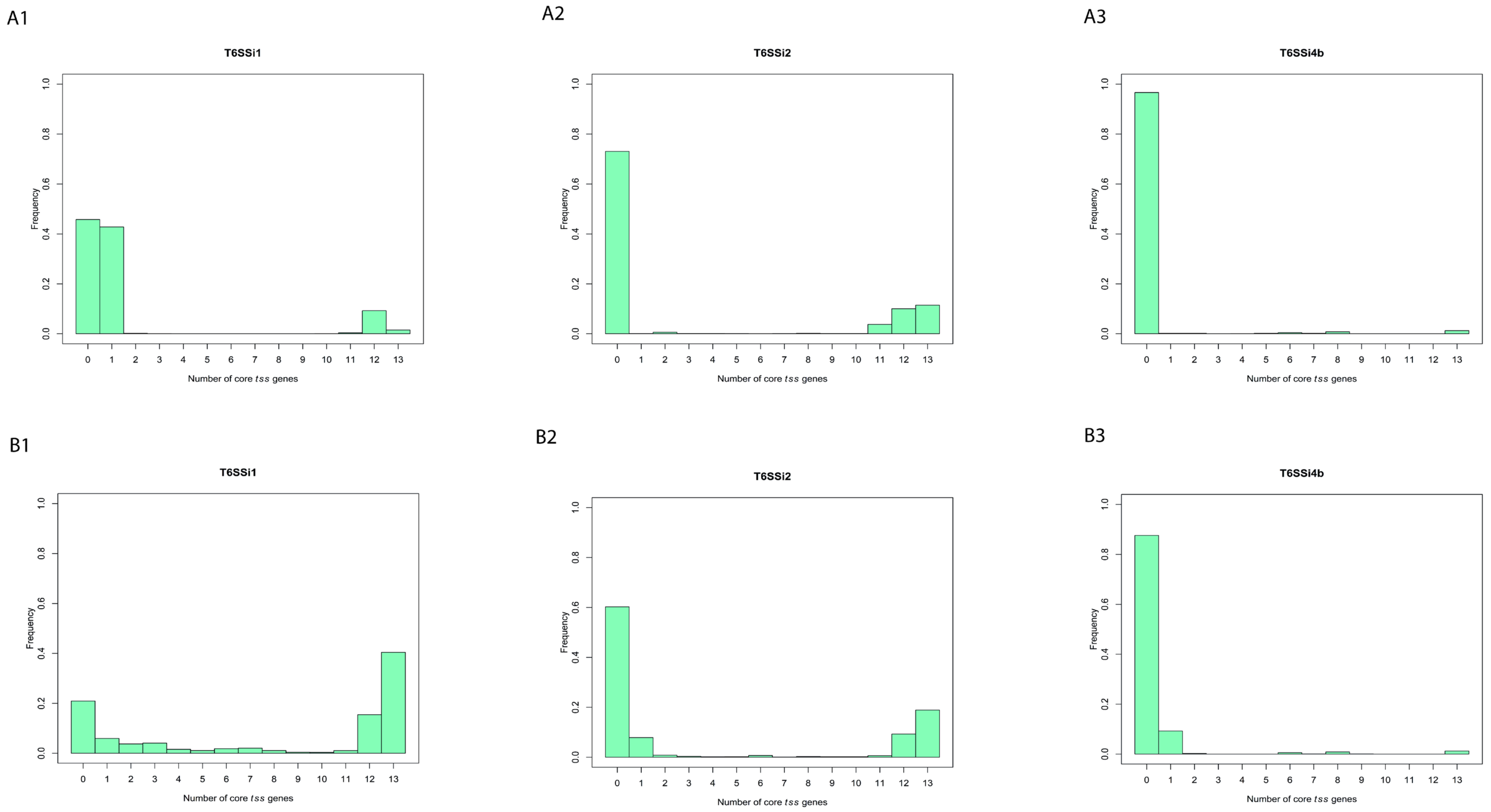

Supplementary Figure S4. Distribution of detected core *tss* genes for both databases used a source for T6SS components. Bar graphs showing the fraction of genomes with the indicated number of core *tss* genes (0-13) in both databases (DB1 **A**; DB2 **B**) for T6SS^{i1} (**A1** and **B1**), T6SS^{i2} (**A2** and **B2**) and T6SS^{i4} (**A3** and **B3**).

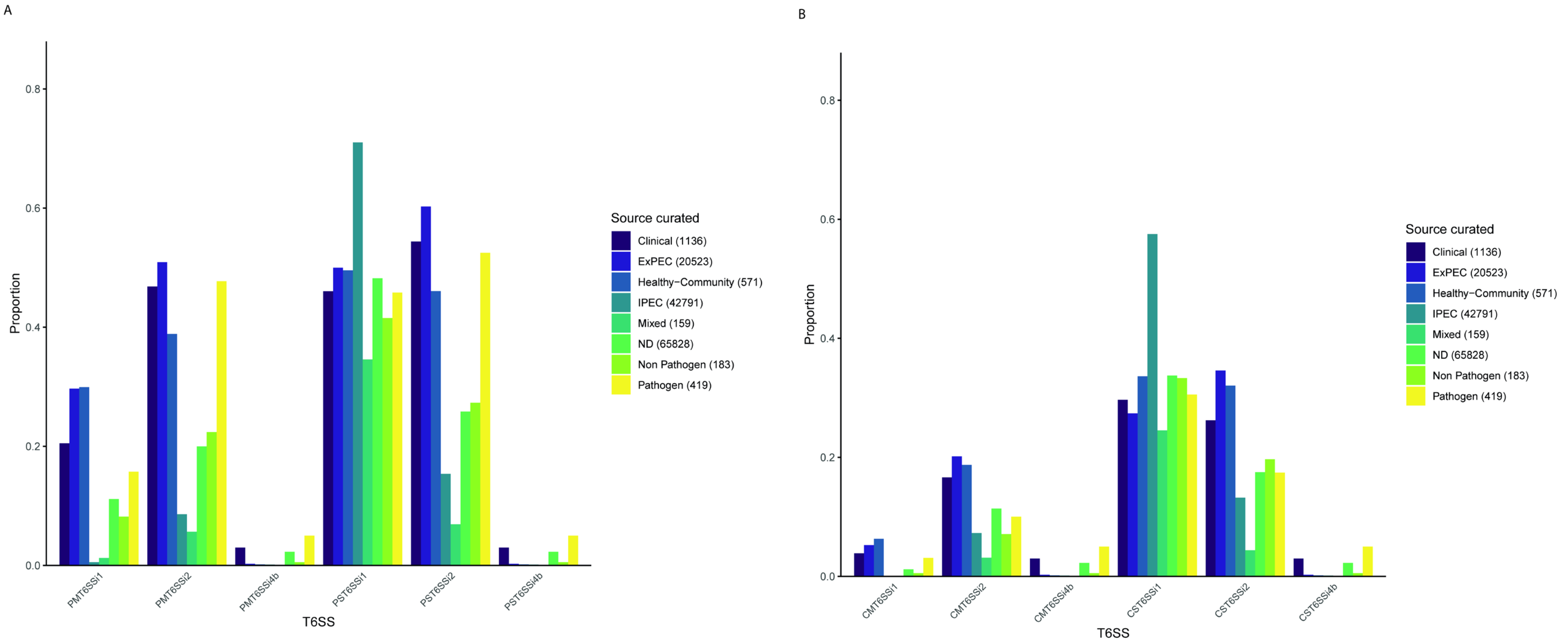

Supplementary Figure S5. Proportions of T6SS[i] subclasses across clinically relevant groups for both databases used a source for T6SS components. Panel **A** contains the proportion of T6SS[i] subclasses that were detected as *Present*, while those that were detected as *Complete* are shown in panel **B.** Each panel contains the proportion of detected T6SS[i] subclasses for both databases, DB1 (M) and DB2 (S).

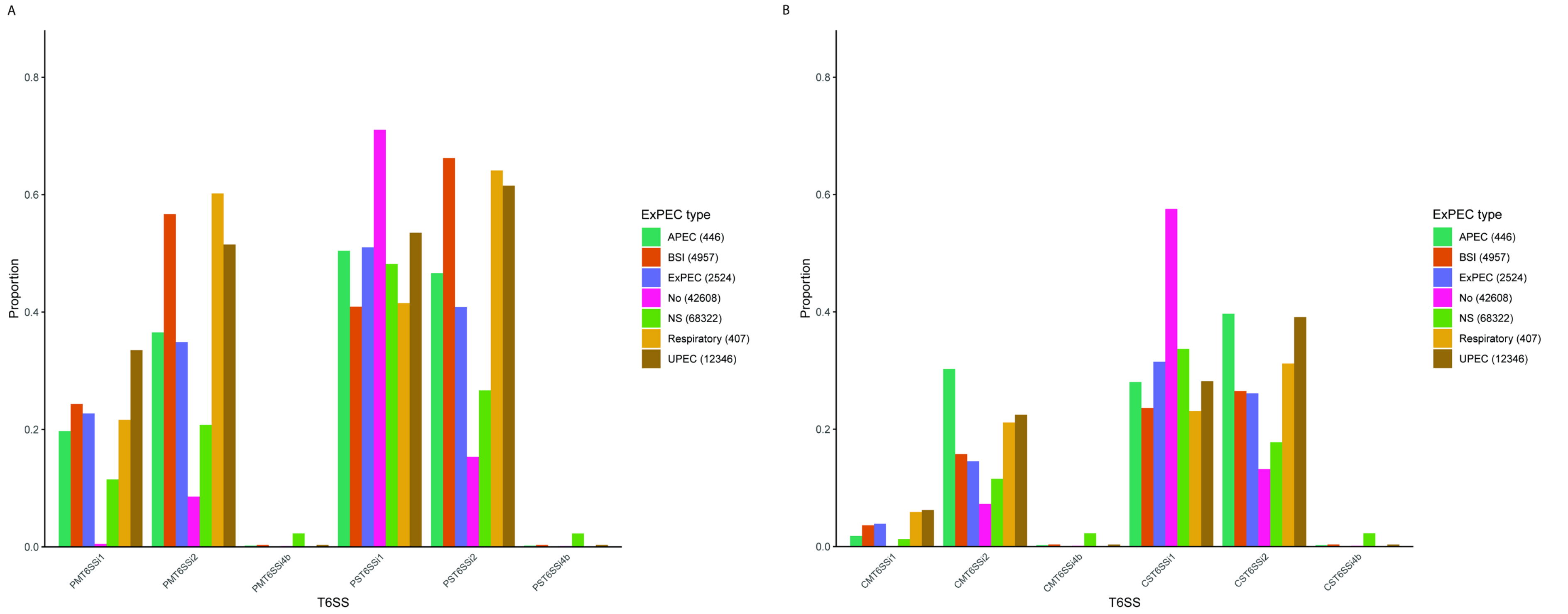

Supplementary Figure S6. Proportions of $T6SS^i$ subclasses in ExPEC type for both databases used a source for T6SS components. Panel **A** contains the proportion of $T6SS^i$ subclasses that were detected as *Present*, while those that were detected as *Complete* are shown in panel **B.** Each panel contains the proportion of detected $T6SS^i$ subclasses for both databases, DB1 (M) and DB2 (S).

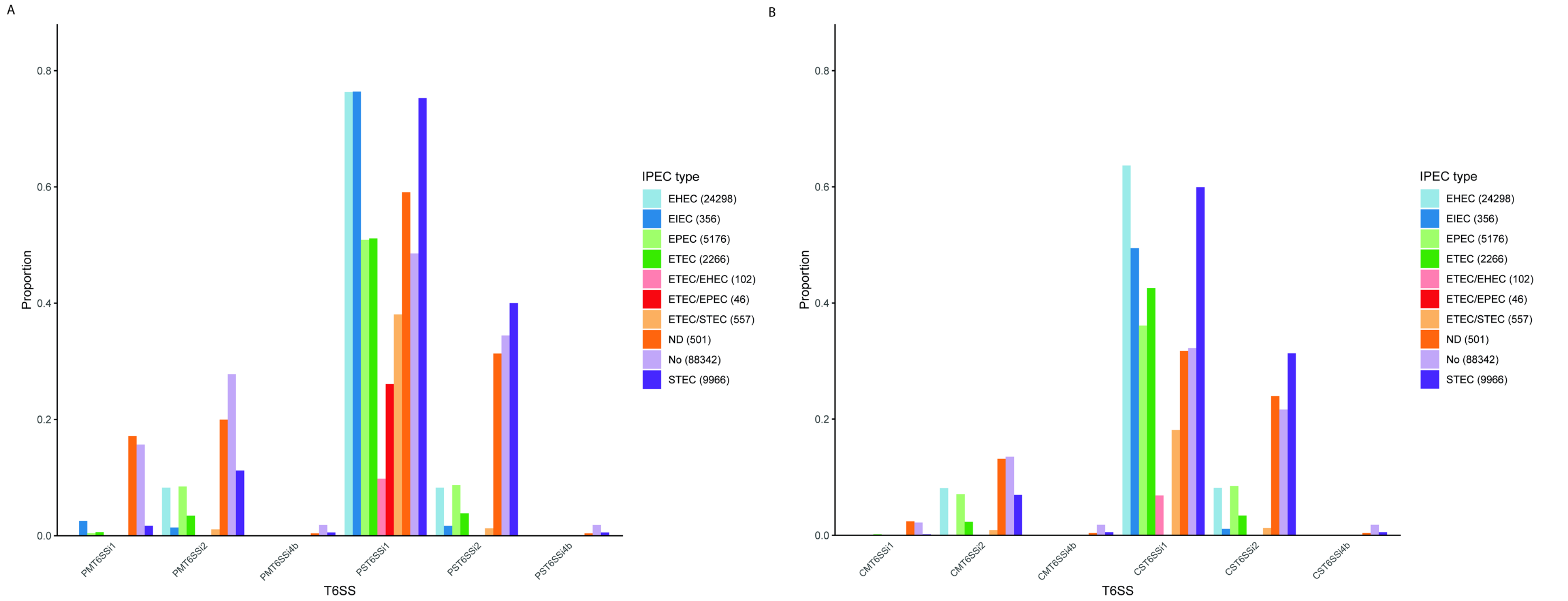

Supplementary Figure S7. Proportions of T6SS^i subclasses in IPEC type for both databases used a source for T6SS components. Panel **A** contains the proportion of T6SS^i subclasses that were detected as *Present*, while those that were detected as *Complete* are shown in panel **B.** Each panel contains the proportion of detected T6SS^i subclasses for both databases, DB1 (M) and DB2 (S).

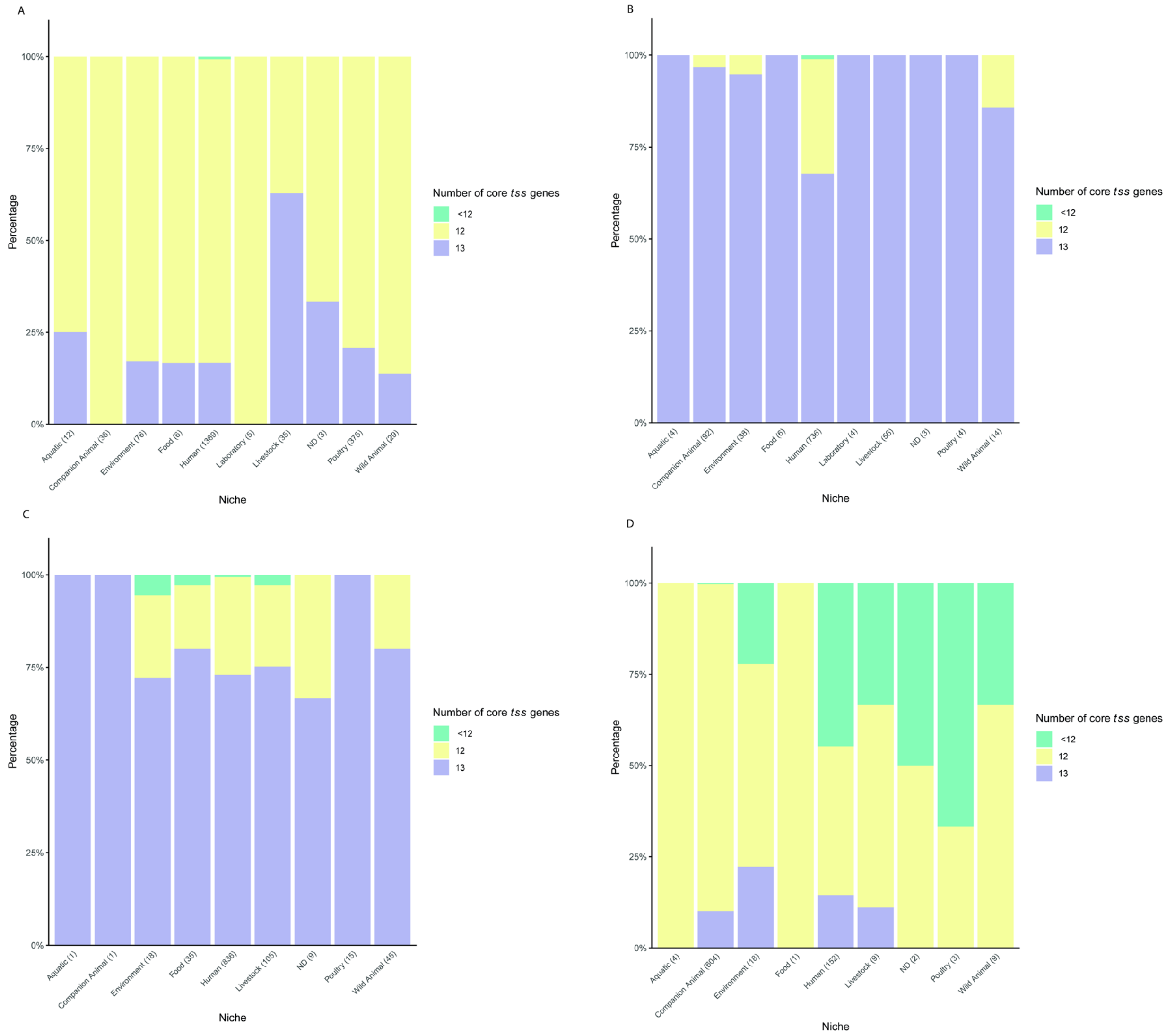

Supplementary Figure S8. Niche patterns for sequence types with truncation of one of core *tss* genes (T6SSi[1]). Bar graphs showing the relative percentage of complete (represented by the detection of 13 *tss* genes) or variants of incomplete (12 or fewer *tss* genes) T6SS[i1] in relation to *Niche* of genomes in ST95 (**A**), ST372 (**B**), ST442 (**C**) and ST167 (**D**).

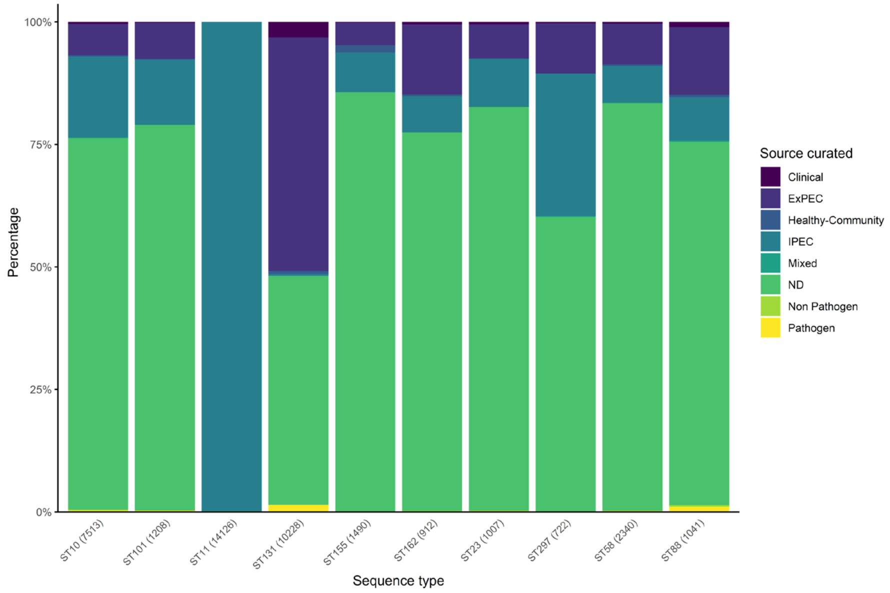

Supplementary Figure S9. Distribution of clinical categories in STs showing a considerable fraction of both IPEC and ExPEC genomes. The bar plot shows the distribution of *Source curated* in ST10, ST101, ST155, ST23, ST297, ST58 and ST88, which all showed an affiliation with both IPEC and ExPEC. ST131 and ST11 serve for comparison as they represent the most dominant ExPEC ST and IPEC ST, respectively.

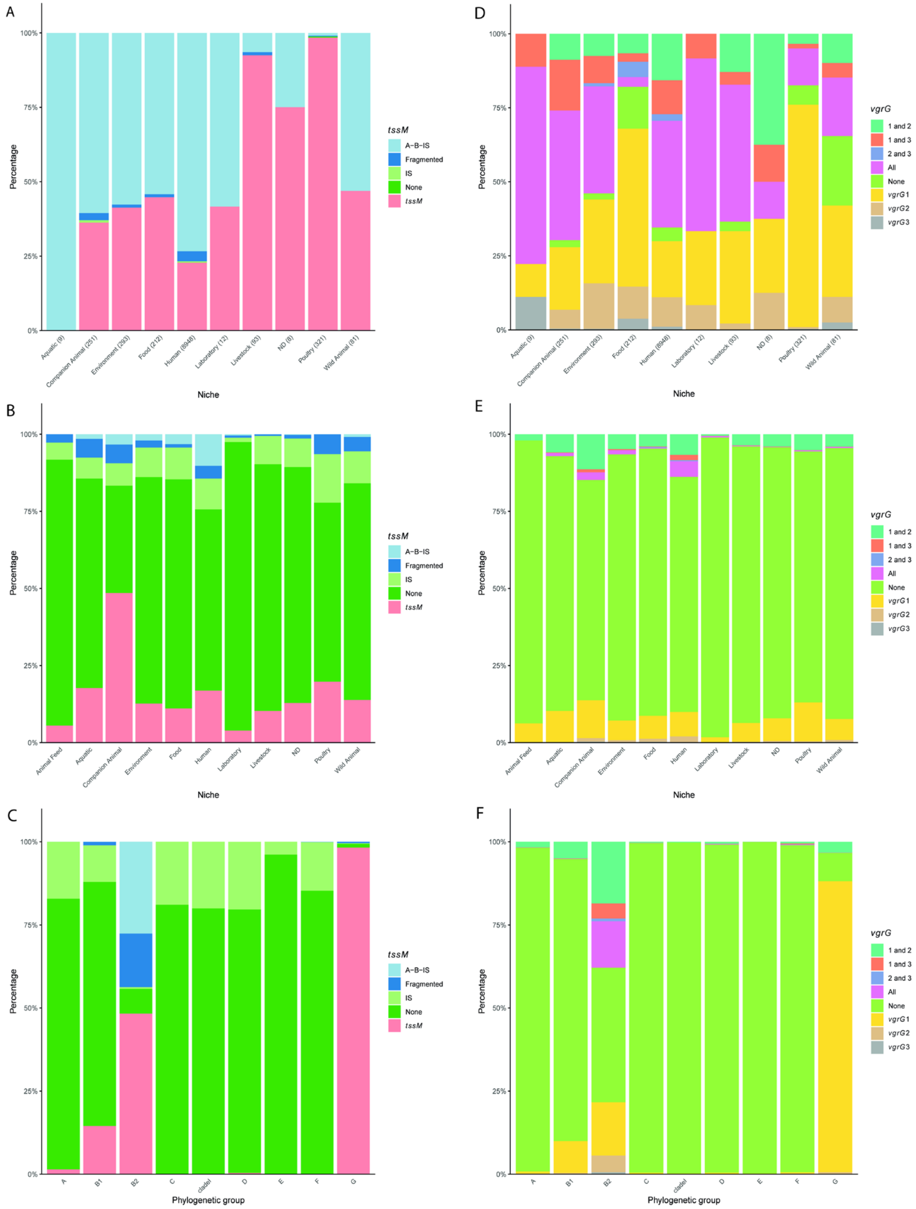

A
B
C
D
E
F
Percentage
Niche
Phylogenetic group
tssM
A-B-IS
Fragmented
IS
None
tssM
vgrG
1 and 2
1 and 3
2 and 3
All
None
vgrG1
vgrG2
vgrG3
0%
25%
50%
75%
100%
Aquatic (9)
Companion Animal (251)
Environment (293)
Food (212)
Human (6948)
Laboratory (12)
Livestock (93)
ND (8)
Poultry (321)
Wild Animal (81)
Animal Feed
Aquatic
Companion Animal
Environment
Food
Human
Laboratory
Livestock
ND
Poultry
Wild Animal
A
B1
B2
C
cladeI
D
E
F
G

Supplementary Figure S10. Differences in *tssM* fragmentation and vgrG variants within ST A. Bar graphs showing the relative distribution of T6SS^{i2}-related *tssM* or its fragments in ST 131 in different niches (same as Fig. 5A). *A-B-IS* corresponds to the described fragmentation by IS*Ec12* (Cummins, 2023) and detection of both fragments (A, B) and the IS, *Fragmented* corresponds to detection of other combinations of fragments, *IS* to detection of only the IS, *None* to no detection of any of the elements, and *tssM* to the detection of an intact *tssM* gene. To provide broader context of *tssM* fragmentation, **B** depicts the same distribution across the whole collection, while **C** provides an overview across phylogenetic groups. **D**. Bar graphs showing the relative distributions of ST131-relevant *vgrG* (here referred to as 1-3) in different niches for ST131 (same as Fig. 5B). To provide broader context of *tssM* fragmentation, **E** depicts the same distribution across the whole collection, while **F** provides an overview across phylogenetic groups.

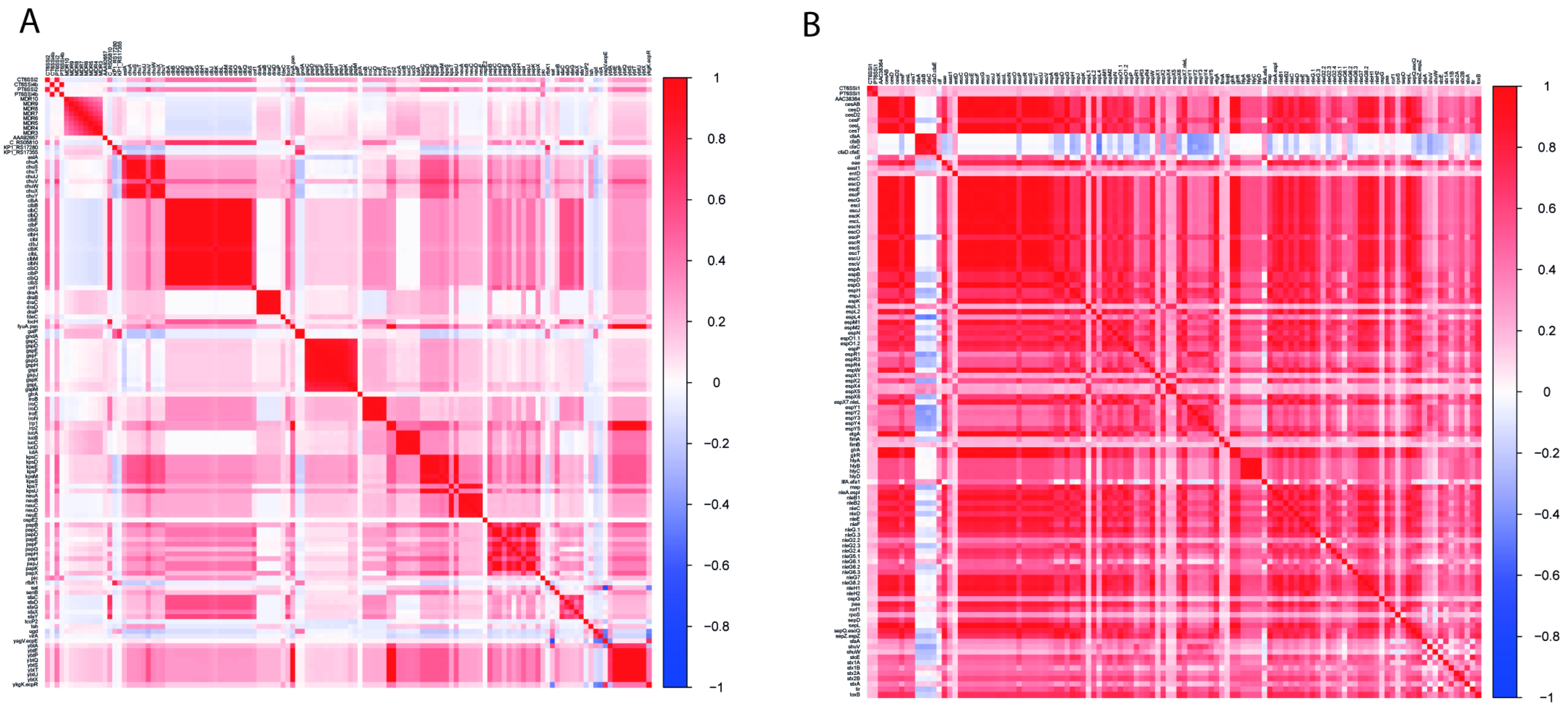

Supplementary Figure S11. ExPEC- and IPEC-associated VAG clusters. A Zoomed-in portion of the clustergram from Fig. 6A highlighting Cluster 1 which contains typical ExPEC VAGs. **B**. Zoomed-in portion of the clustergram from Fig. 6A, highlighting Cluster 2 which contains typical IPEC VAGs.

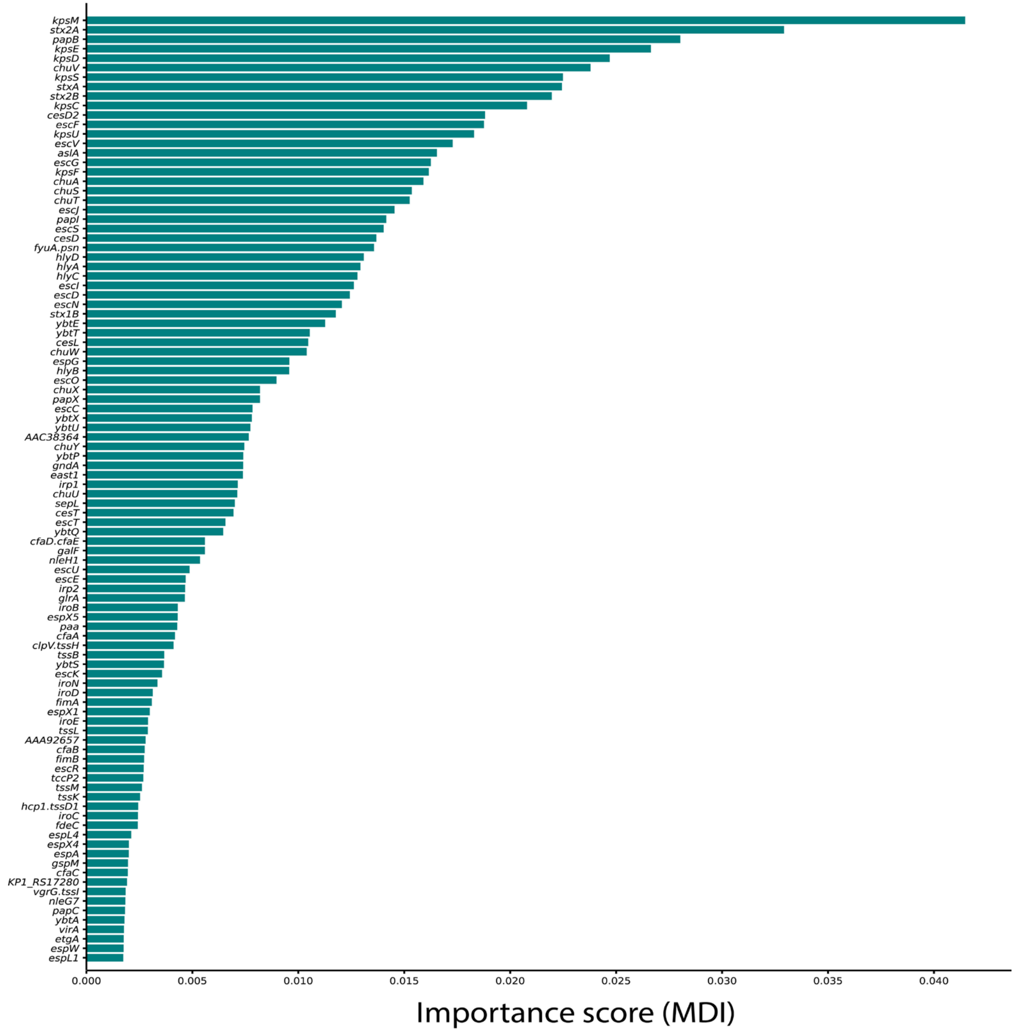

Supplementary Figure S12. **Extra Trees model feature importance**.
Barplot highlighting the importance score of 100 of the 262 different features (VAGs and T6SS presence/absence) used in training the Extra Trees model that achieved the highest accuracy.

Supplementary Table S1. Original EnteroBase metadata datasets (Assembly stats, Phylotype, and Annotation), metadata added or curated in this study (columns: **Source.curated**, **ExPEC.type**, **IPEC.type**, **Source.Pathogen**, **Source.Pathogen.details**, **Gender**, **ExPEC_STs**), and results of our screen for T6SS^{i} and VFDB.

Supplementary Table S2. Core *tss* genes references and division into T6SS^{i} subclasses for DB1 (Ma, 2013 and KO_178_B) and DB2 (SecReT6). For DB1, an additional reference from GenBank or SecReT6 was added, given that the original gene naming did not reveal the *tssA-tssM* annotation.

Supplementary Table S3. Prevalence of T6SS^{i} subclasses (*Presence* and *Completeness*) across selected metadata categories and 35 prevalent STs (overall and separately for DB1 and DB2). **PT6SSi1-i4b** corresponds to the *Presence* of specific T6SS^{i} subclasses, while CT6SS**i1-i4b** corresponds to *Completeness*. **P/C-multiple** corresponds to genomes with multiple T6SS^{i} subclasses. **N** specifies the number of genomes in the respective categories.

Supplementary Table S4. Sequence types recovered for each category of ExPEC type (APEC, BSI, Respiration, UPEC and ExPEC-other). **N** represents the number of genomes detected for given ST.

Supplementary Table S5. Sequence types recovered for main categories of IPEC type (EHEC, EIEC, EPEC, ETEC and STEC)**. N** represents the number of genomes detected for given ST.

Supplementary Table S6. Sequence types recovered for the ExPEC category from Source curated. **N** represent the total number of genomes recovered for a given ST, **% of all ExPEC** refers to the percentage of a given ST among all the ExPEC labelled genomes, **ExPEC fraction for ST** shows the fraction of ExPEC-linked genomes to the total number of genomes for a given ST for the 39 most dominant ExPEC STs, **ST_total** specifies the total number of genomes for STs with calculated ExPEC fraction.

Supplementary Table S7. Sequence types recovered for IPEC category from Source curated. **N** represent the total number of genomes recovered for a given ST, **% of all IPEC** refers to the percentage of a given ST among all the IPEC labelled genomes, **IPEC fraction for ST** shows the fraction of IPEC-linked genomes to the total number of genomes for a given ST for the 39 most dominant IPEC STs, **ST_total** specifies the total number of genomes for STs with calculated IPEC fraction.

Supplementary Table S8. P-values calculated using the pairwise Wilcoxon test with Benjamini-Hochberg (BH) adjustment at significance level of α = 0.05. The compared categories are specified in the first column, **P_value** represent value before the BH adjustment, while **Adjusted_P_value** represents the final one. Value 0 represents extremely low p-values, which provide strong evidence against the null hypothesis (below the smallest value the software can represent); in the text, the value is then presented as $<10^{-200}$; value NA represents that categories could not be compared together.

Supplementary Table S9. Correlation coefficients calculated using polychoric correlation coefficient for $T6SS^i$ subclasses *Completeness* and *Presence*, MDR (defined on multiple levels) and 227 VAGs from VFDB.

Supplementary Table S10. Prevalence of $T6SS^i$ regulatory genes for *Source curated*.

Supplementary Table S11. Correlation coefficients calculated using polychoric correlation coefficient for $T6SS^i$ subclasses *Completeness* and *Presence* and $T6SS^i$ regulatory genes.

Supplementary Table S12. Performance evaluation of eight models used to predict whether genomes are IPEC or ExPEC.

Supplementary Table S13*. Source Predicted* indicates which of the ND-labeled genomes are ExPEC-like (**ND_ExPEC**) or IPEC-like (**ND_IPEC**) based on our Extra Trees model.

Supplementary Table S14. Performance evaluation of eight models when a third category of *Commensal* is introduced, illustrating the poor scores for this category (third value in columns *Precision*, *Recall* and *F1-Score*, highlighted in bold).
